# Half a degree and rapid socioeconomic development matter for heatwave risk

**DOI:** 10.1038/s41467-018-08070-4

**Published:** 2019-01-11

**Authors:** Simone Russo, Jana Sillmann, Sebastian Sippel, Monika J. Barcikowska, Claudia Ghisetti, Marek Smid, Brian O’Neill

**Affiliations:** 1 European Commission, Joint Research Centre, Ispra, 21027 Italy; 2 Institute for Environmental Protection and Research (ISPRA), Rome, 00144 Italy; 3 Center for International Climate and Environmental Research (CICERO), Pb. 1129 Blindern, N-0318 Oslo, Norway; 4 Norwegian Institute of Bioeconomy Research, 1431 Ås, Norway; 5 Environmental Defense Fund, New York, 10010 USA; 6 NOVA IMS, Universidade Nova de Lisboa, Lisbon, 1069-061 PT Portugal; 7 University of Denver, Denver, CO 80208 USA

## Abstract

While every society can be exposed to heatwaves, some people suffer far less harm and recover more quickly than others from their occurrence. Here we project indicators of global heatwave risk associated with global warming of 1.5 and 2 °C, specified by the Paris agreement, for two future pathways of societal development representing low and high vulnerability conditions. Results suggest that at the 1.5 °C warming level, heatwave exposure in 2075 estimated for the population living in low development countries is expected to be greater than exposure at the warming level of 2 °C for the population living in very high development countries. A similar result holds for an illustrative heatwave risk index. However, the projected difference in heatwave exposure and the illustrative risk index for the low and very high development countries will be significantly reduced if global warming is stabilized below 1.5 °C, and in the presence of rapid social development.

## Abstract (web summary)

Climate extremes, exposure and vulnerability all contribute to global difference in heatwave risk. Here the authors investigated the inequality in global heatwave risk under both 1.5 and 2 °C scenarios and found that heatwave risk for the poor under 1.5 °C scenario exceeds that risk for the rich under 2 °C scenario.

## Introduction

The large socioeconomic costs of heatwaves make them a crucial target for impact assessments of climate change scenarios. Recent studies have focused on changes in the frequency, intensity, and duration of extreme events that affect their risk to human society^1–6^, in some cases differentiating the occurrence of those hazards in low income versus high income countries^7,8^. According to the Intergovernmental Panel on Climate Change^9,10^ climate change risks are determined not only by climate extremes (the hazards) but also by the exposure and vulnerability of society to these hazards^10,11^. Here, we analyze and discuss changes in heatwave hazard, population exposure, and a vulnerability proxy. Subsequently, we derive an illustrative heatwave risk index (IRI) as the product of the probability of its occurrence (hazard) and normalized levels of exposure and a proxy for vulnerability^12^ (see Eq. (1)).

We calculate the IRI at two different levels of warming (1.5 °C, 2 °C) and for two alternative scenarios of societal development based on the Shared Socioeconomic Pathways (SSPs)^13^ designed to explore a range of exposures, potential vulnerabilities and potential capabilities to adapt to climate change. In particular, SSP1 corresponds to a society with low population growth and rapid social and economic development (low vulnerability), whereas the SSP4 represents a future society with high population growth in currently high fertility countries and a high degree of inequality (high vulnerability)^13^.

As metric for heatwave hazard we use the decadal probability of experiencing an extreme heatwave. A heatwave is defined using the Heat Wave Magnitude Index daily^14^ (HWMId), which takes into account both duration and temperature anomalies of a heatwave into a single number. Extreme heatwaves are those that occur on average every five hundred years under present climate conditions (hereafter HW500Y; results for 100-year return heatwaves are shown in Supplementary). The hazard is estimated through extreme value analysis using a block maxima approach^1,15,16^, based on multi-model ensemble simulations (four models, each with 1000 years members or more) provided by the Half A degree additional warming, Prognosis and Projected Impacts (HAPPI) project for the present climate and at warming levels of 1.5 and 2 °C (see Methods).

Following recent studies^8,17,18^, we combine the projected heatwave hazard with projections of spatially explicit population density consistent with the SSPs^13,19^ to calculate exposure. Calculations of risk usually combine exposure to a particular hazard with dose-response relationships relating exposure to an outcome of interest, such as mortality or morbidity due to heatwaves. These relationships reflect the level of vulnerability of the exposed population. Lacking such dose-response relationships for heatwaves that are applicable globally, we instead adopt the Human Development Index^20^ (HDI) as an indicator of broadly defined vulnerabilility. The HDI is a composite indicator introduced by the UNDP in 1990 to assess the socioeconomic development of countries. Other studies have used Gross Domestic Product (GDP) to account for vulnerability to climate change^8,21–23^; HDI is a more comprehensive measure than GDP as it takes income, health, and education into account. Low and very high-human-development countries are defined by using the fixed cutoff points based on quartiles of HDI values introduced by the 2014 Human Development Report (*HDI* < 0.55 and *HDI* > 0.8, respectively (see HDR_technical_notes.pdf and Methods). HDI has been shown to outperform several more recent indices as a generic national-level index of social vulnerability to climate change^24^. HDI also shows high significant correlation with historical measures of country vulnerability to climate change such as the Notre Dame-Global Adaptation Initiative Country Index (ND-GAIN)^25^ (see 'HDI versus other vulnerability indices' section). However, it is important to emphasize that HDI can neither serve as a specific (or causal) vulnerability measure to heatwaves or any other climate hazard, nor does it indicate adaptive capacities to specific heatwaves per se. We use recent projections of HDI for all countries through 2075, consistent with the demographic, economic, and education assumptions in the SSPs^26^ in order to calculate the IRI in a manner that illustrates how vulnerability can affect risk, not to estimate actual heatwave risk outcomes.

Here, we derive IRI to illustrate relative composite spatial patterns of hazard, exposure, and vulnerability at the global scale rather than definitive or quantitative risk estimates. We calculate normalized and non-normalized versions of IRI. In the normalized IRI, present and projected HDI and population density variables are transformed to the same range of variability before aggregation by normalizing in (0, 1) using the Johnson Cumulative Distribution Function^27^ (CDF) fitted to the present period (see Methods). Normalized IRI thus represents the probability of occurrence of an extreme heatwave (HW500y) scaled by normalized population density and level of social development. An IRI of zero indicates low or negligible risk relative to the other locations, for instance due to very low population density and thus low exposure, or very high HDI and thus low vulnerability. In the normalized version, an IRI of 1.0 represents the highest possible level of risk. In the present period, due to the construction of normalized IRI, its values lie between zero and the present-day hazard probability (i.e., a HW500y in a present decade has a chance of 0.2%). In the future, risk can—in principle—either increase or decrease as its components (hazard, population, and HDI) increase or decrease, and these changes will be reflected in the IRI. For example, if hazard probability and HDI remain constant, but population density decreases, IRI would decrease. In contrast, very large increase of IRI in a future period might reflect increase in the hazard probability, or—rather theoretically—an increase in population by several orders of magnitude. In summary, IRI explores relative effects of hazard probability, exposure, and vulnerability. IRI values are not based on or calibrated to a dose-response relationship, and hence, the normalized IRI does not preserve physical units. Accordingly, due to the lack of a physical relationship, the IRI (normalized) approach implicitly assumes that relative changes in hazard probabilities, exposure, and vulnerability of the respective normalized distributions are equally important. Consequently, IRI values cannot be interpreted in terms of physical or quantitative risk estimates.

## Results

### Heatwave hazard

**Fig. 1:**
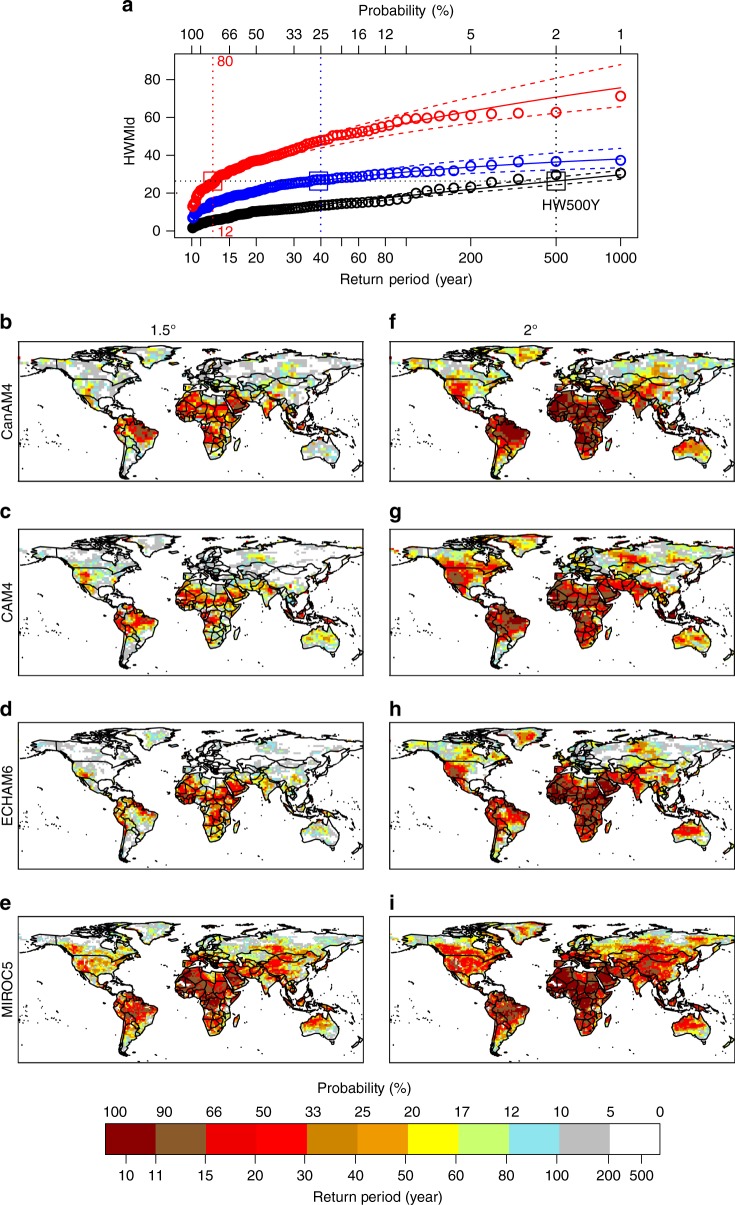
Probability of occurrence of extreme heatwave. **a** Extreme value analysis GEV-fit for decadal-maximum HWMId at a location in Central Africa Republic (18.75°E, 4.69°N) as a function of return period (bottom *x*-axis) and hazard (upper *x*-axis expressed as decreasing probability) at present climate (black curves), 1.5 °C (blue curves) and 2 °C (red curves) warming levels. The colored dashed curves give the 95%-confidence interval, based on a likelihood estimates (see method), and the open colored circles are the simulated decadal-maximum HWMId values. The open squares represent the 500-year return level heatwave (HW500y) in present climate (black) and at 1.5 and 2 °C level of warming, blue and red, respectively. **b**–**i** Spatial distribution of the probability and return level of a heatwave with five-hundred years return period under 1.5 °C (**b**–**e**) and 2.0 °C (**f**–**i**) warming for multiple models

Figure 1b–i depicts the spatial distribution of the HW500Y hazard, expressed in terms of probability of occurrence and the corresponding return period at the 1.5 and 2 °C warming levels, occurring at least once every 100 years over most of the land surface, and radically increasing across Africa, Middle East, and and parts of Southeast Asia and Latin America to at least once per decade (Fig. 1f–i). Substantial changes in heatwave frequencies in these regions are related to lower year-to-year temperature variability, and thus a higher warming-to-noise ratio leading to larger relative changes^28^. Similar changes in frequency are shown for heatwaves occurring every one hundred year in the present period (see Supplementary Fig. 1). Under the 1.5 °C scenario, the frequency of HW500Y events is substantially reduced (relative to 2 °C warming), with maximum frequencies reduced to once every several decades.

### Heatwave exposure

**Fig. 2:**
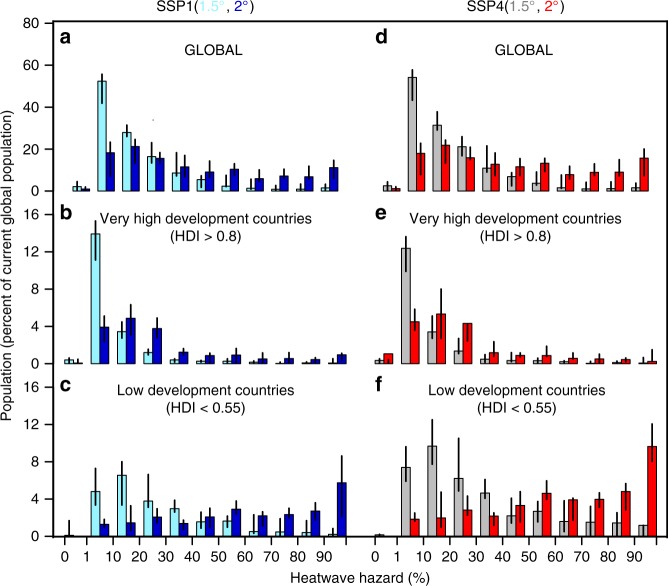
Population exposure to heatwave hazard. **a** Bar plots show the ensemble model median, with associated range represented by black lines, of the global population in 2075 exposed to different probabilities of HW500Y events occurring in a given decade at 1.5 °C(gray bars) and 2 °C(red bars) warming and under the SSP1 pathway. Population in 2075 is expressed as a percentage of the current global population. The bar plots are calculated for all the grid points of the global domain with population density greater than 0. **b**, **c** as for a, but for very high and low-human-development countries with HDI > 0.8 and HDI < 0.55, respectively. **d**–**f** as **a**–**c**, respectively, but for the SPP4 scenario

Population exposure to the heatwave hazard is affected not only by these changes in frequency but also by projected population changes. By the end of the century, the global population is expected to reach approximately 120 and 140% of the present population in SSPs 1 and 4, respectively. In addition, in both SSPs more population growth occurs in countries currently at lower levels of development, and, as we have already noted, increase in the heatwave hazard are larger in those countries as well. As a consequence, exposure (the product of the hazard and population exposed to it) increases most in countries at lower levels of development. In fact, we find that at the 1.5 °C level the population in the low-human-development countries (defined as HDI < 0.55) will be exposed to equal or greater levels of heatwave hazard than the population in very high-human-development countries (defined as HDI > 0.8) under the 2 °C scenario (see Fig. 2). A list of low and very high-human-development countries, a grouping introduced by the UN Development Program and assigned here, according to the 2015 HDI values, is reported in Supplementary Table 1. Exposure is higher not only because of the difference in hazard, but also because the population exposed at the end of the century is larger in low-human-development countries, equivalent to 25 and 39% of present global population in SSPs 1 and 4, respectively, compared to 20 and 18% in the very high-development-countries.

### Heatwave risk

**Fig. 3:**
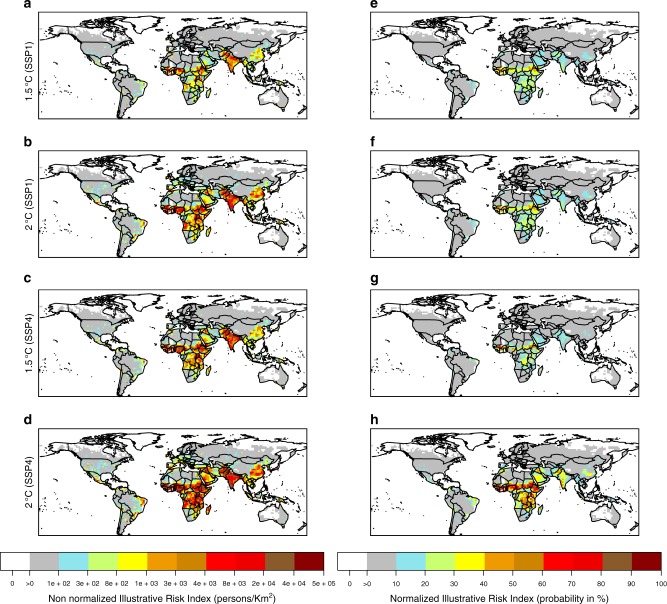
Spatial distribution of the Illustrative risk index. **a**–**d** Non Normalized IRI for the ECHAM6 model: the values at each grid point are calculated as the product of the probability of occurrence of HW500Y the value of population density, and one minus the Human Development Index (see Methods). **e**–**h** Normalized IRI for the ECHAM6 model: the values at each grid point are calculated as the product of the probability of occurrence of HW500Y and the normalized distribution of exposure (population density) and vulnerability (one minus the Human Development Index, see Methods)

The IRI goes beyond exposure to illustrate how accounting for vulnerability could potentially change the outlook for future risk. HDI increases over time in all countries, but at different rates, and therefore vulnerability generally decreases, ameliorating changes in future risk at different rates across countries and scenarios. Projections of the spatial distribution of non-normalized IRI based on one representative climate model (Fig. 3a–d), when compared to projections of the hazard alone using the same model (Fig. 1, panel for ECHAM model), show that the consideration of population density and an index of vulnerability substantially changes the outlook for potential risk. The IRI in North America, most of Latin America, Australia, and much of Europe is substantially muted, relative to the rest of the world, to a degree that is not evident in the projection of the heatwave hazard. In contrast, the IRI in South and East Asia is on par with the relatively high values in Sub-Saharan Africa, despite having a relatively lower heatwave hazard in those areas.

Given the fact that population density can range much more widely than the value of HDI, the scale of the non-normalized IRI is influenced mainly by variability in population density. The normalized IRI transforms the three variables into standard uniform units (see Methods). It produces a similar spatial pattern of the IRI to the non-normalized version (Fig. 3e–h), but with a smaller index value in South and East Asia relative to other locations due to the more limited effect of population density on IRI after normalization (normalizing only HDI, and not population, does not produce this effect, see Supplementary Fig. 2). Because the probability of HW500y is likely to increase substantially in 1.5 or 2 °C worlds (see example in Fig. 1), while projected changes in exposure or vulnerability are not as large in relative terms, changes in the normalized IRI will be to a large extent driven by changes in the hazard component.

Other analyzed HAPPI models show similar patterns in the spatial distribution of normalized IRI (Supplementary Figs. 3 and 4).

**Fig. 4:**
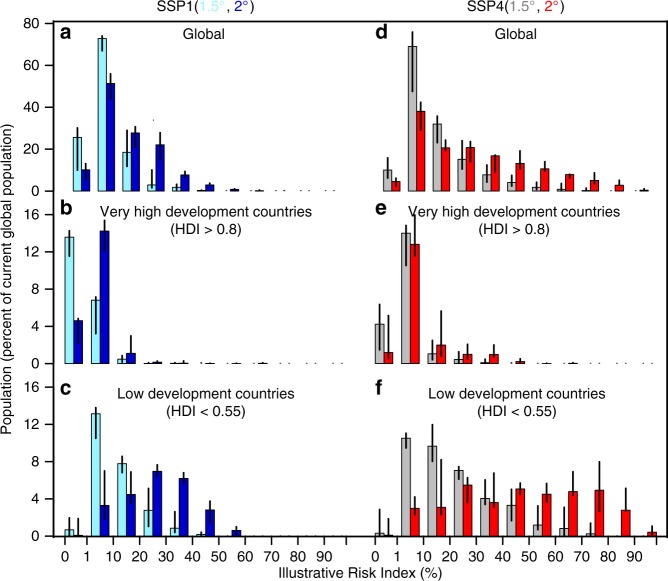
Population as function of IRI. **a** Bar plots show the ensemble model median, with associated range represented by black lines, of the population in 2075 (measured as percent of current global population) that experiences different IRI levels at 1.5 °C(gray bars) and 2 °C(red bars) warming, and under the SSP1 pathway. The bar plots are calculated for all the grid points of the global domain with population density greater than 0. **b**, **c** as for **a**, but for population in countries with HDI > 0.8 and HDI < 0.55, corresponding to very high and low-human-development countries, respectively. **d**–**f** as for **a**–**c**, respectively, but for the SPP4 pathway

The value of IRI is highest in the SSP4 scenario with 2 °C warming (Fig. 4). Under these circumstances, a population equivalent to 77% of the current global population will experience an illustrative heat risk value greater than 20% (Fig. 4d). In low developement countries a population equivalent to 27% of the current global population, the IRI value will be greater than 50% (see Fig. 4f). Values of IRI are lowest in the SSP1 scenario with 1.5 °C warming. In that case, IRI nowhere reaches values above 50%, and in low-development-countries a population equivalent to only 5% of the present global population experiences IRI values greater than 20% (Fig. 4c).

**Fig. 5:**
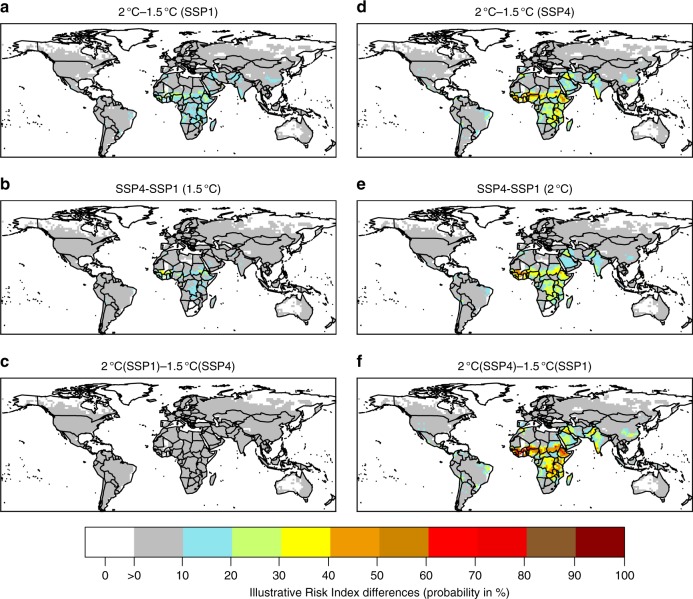
Differences in Normalized IRI. Normalized IRI differences are calculated at each grid point by means of IRI values for the ECHAM6 model. **a**, **d** Differences of IRI between 2 and 1.5 °C under the low vulnerability (SSP1) and high vulnerability (SSP4) scenarios, respectively. **b**, **e** Differences of IRI between SSP4 and SSP1 for the 1.5 and 2 °C warming levels, respectively. **c** Differences of IRI between 2 °C under the SSP1 scenario and 1.5 °C under the SSP4 scenarios. **c**, **f** Differences of IRI between the most pessimistic (2 °C level of warming under the SSP4 scenario) and most optimistic (1.5 °C level of warming under the SSP1 scenario) scenarios

It follows then that the greatest reductions in IRI are achieved by both limiting warming to 1.5 °C and fostering rapid social development (SSP1), particularly across sub-Saharan Africa (Figs. 3a, e and 5f) where most of the present low-human-development countries are located (Supplementary Fig. 5d). Differences between the normalized IRI values across other scenario combinations show that the risk index increases in all inhabited regions if global warming reaches 2 °C rather than being limited to 1.5 °C, and if the degree of exposure and the vulnerability proxy (HDI) of future society follows SSP4 instead of SPP1 (Fig. 5, see Supplementary Figs. 5–7 for other models). The effect of differences in climate and development also interact. For example the impact of the additional half a degree warming on the illustrative risk index is substantially amplified under SSP4 compared to SSP1 (see Fig. 5a, d). In addition, different effects on IRI of climate and societal factors implies that in this illustrative calculation, the consequences of 2 °C warming in SSP1 are similar to those of 1.5 °C warming in a more vulnerable society (SSP4) (see Fig. 5c). The comparison of Fig. 4b, c, e, f, suggests also a prominent contrast between the impact of global warming on the very high and low-human-development countries. For example, the IRI levels in very high human-development-countries remain low (values less than 20% almost for all population) even with 2 °C warming in SSP4 (Fig. 4e). In contrast, in low-human-development countries under the same inequality scenario (SSP4), the IRI level is almost always above 10% even at a warming level of 1.5 °C (Fig. 5f). More generally, the illustrative heatwave risk index for the population living in low-human-development countries at the 1.5 °C warming level is typically larger than the values for the very high-human-development countries, even with 2 °C warming. Amplified patterns in heat extremes, i.e., the hazard component, for countries with low human development, had been pointed out earlier^28^, and thus our results appear consistent with previous literature. The analysis repeated for the heatwaves defined with one hundred years return levels, i.e., HW100Y, shows similar results (see Supplementary Fig. 8), as does an analysis without using normalized HDI values (see Supplementary Fig. 9).

## Discussion

In this study, we have quantified heatwave hazard, exposure and a vulnerability proxy, associated with a global warming stabilized at 1.5 and 2 °C levels compared to preindustrial climate conditions. In addition, we have presented and discussed the aggregation of the three dimensions as an illustrative risk index (IRI). The results were also differentiated between two socioeconomic pathways, which represent either rapid social and economic development (SSP1) or high inequality (SSP4) by the end of the century, and which strongly contrast in exposure and vulnerability.

The analysis highlights a stark contrast in the aggregated risk metric between low and very high-human-development countries, quantified for different combinations of warming levels and socioeconomic pathways.

Even under the 1.5 °C warming level, the low-human-development countries (representing future populations equal to 25 or 39% of the present global population in the SSP1 and SSP4, respectively) experience exposure levels equal to or greater than the levels for the very high-human-development countries with 2 °C warming. We also find that, in agreement with a recent study^8^, holding the temperature below 1.5 °C warming yields a large potential to reduce the levels of the heatwave exposure. Results for the IRI suggest that the same could be true for heatwave-related risks to society, especially for low-human-development countries. In addition, we show that the IRI values can be reduced, not only by limiting global temperature increase to 1.5 °C, but also with rapid socioeconomic development.

The role of the latter might be crucial, considering that some studies estimate the likelihood of reaching the Paris agreement targets, i.e., stabilizing warming at the 1.5 or 2 °C, to be low (approximately 5% and 10%, respectively)^29^.

This work represents an initial attempt to quantify differences in heatwave hazard, exposure and illustrative risk between different warming levels and socioeconomic pathways that is global in scope. An important caveat to the study is that our illustrative risk index does not use a dose-response relationship relating exposure to a specific heatwave-related impact. Rather, it uses a proxy for vulnerability, the HDI, which is a general measure of vulnerability to a wide variety of climate impacts, is not resolved below the level of individual countries, and is not tailored specifically to risks from heatwaves, nor calibrated to specific outcomes such as mortality^30^, morbidity, or reduced labor productivity. Relative changes in any of its components would equally contribute to changes in IRI. Hence, the IRI cannot be interpreted in terms of a physical risk estimate (such as probability of a specific harmful consequence). Relative changes in IRI values across countries, or across scenarios, would be different if a different proxy for vulnerability were used, or a different approach taken to the calculation of the index. Furthermore, a very small hazard probability in the present period leads to large relative changes in the hazard component, which likely dominate changes in IRI. Projections presented here of the heatwave hazard and exposure to it can be interpreted more directly as the distribution of population by the likelihood of experiencing the hazard, but have the shortcoming of not accounting for the differential levels of vulnerability across populations. The IRI illustrates how the incorporation of vulnerability-related information could change outcomes. Future work could also improve on this analysis by accounting for the large heterogeneity in vulnerability within countries. Nonetheless, the incorporation of projections of population exposure and a proxy of human vulnerability to climate-related hazards, such as heatwaves, provides relevant information for global-scale impacts and risk assessments, and points toward ways in which analyses of a wide range of climate risks could be strengthened.

## Methods

### Data

Heatwave magnitude is estimated by means of the Heat Wave Magnitude Index daily^14,31^ (HWMId). Results are tested by comparing the HWMId values with the the annual maximum of 5-day average of daily maximum temperature (TX5x). The HWMId and the TX5x are calculated for the present climate and at 1.5 and 2 °C warming from daily maximum temperature from the HAPPI (Half A degree additional warming, Prognosis and Projected Impacts) project, based on the atmospheric components of the CMIP5 models forced by prescribed Sea Surface Temperature (SST) and sea ice concentrations^32,33^. A recent study^34^ has shown that, particularly over the tropics and Australia, estimates of the changes in the odds of annual temperature extremes can be up to more than a factor of 5 to 10 larger using prescribed SSTs than when using a fully coupled model configuration. This is because the variability of the distribution of annual maximum temperatures, simulated by using prescribed SST, is underestimated with respect to the one simulated by fully coupled model configuration. While this issue can be alleviated to a certain degree by using metrics that are standardized relative to its variability (interquartile range) such as HWMId, findings should still be interpreted as conditional on the period in which sea surface temperatures were prescribed^34^.

Hence, extreme events obtained from these simulations can be seen as conditional on a certain decade, but it is important not to interpret 500-year return periods at multi-decadal scale, precisely because of long-term variability^34^. However, heatwave magnitudes corresponding to this long return period should be representative of extreme heatwaves such as the one in Central Europe in 2003 and in Russia in 2010^14,35^, if a sea surface state corresponding to such an event occurred in the respective decade used to run the ensemble simulations. As prescribed by the HAPPI protocol, the 1.5 and 2 °C simulations use the same aerosol forcing. It is important to emphasize that aerosol emission for the stabilization scenarios are reduced from present days^1,36^. This could produce some differences between heatwave return levels in the stabilized scenarios and present day. However, this does not affect our results that focus on the differences between the two stabilized scenarios. We use four out of the five available simulations that have at least one thousand year runs: Model-1 is the Canadian Fourth Generation Atmospheric Global Climate Model (CanAM4) contributed by the Canadian Centre for Climate Modeling and Analysis^37^. Model-2 is the NCAR-DOE Community Atmosphere Model version 4 (CAM4) coupled to the Community Land Model version 4 (CLM4) with simulations contributed by ETH Zurich^38,39^. Model-3 is ECHAM6.3^40^,contributed by the Max Planck Institute of Meteorology, Hamburg, Germany (global 1.875 °C grid). Model-4 is a high resolution (global 0.5 °C grid) model, contributed by the National Institute for Environmental Studies, Tsukuba, Japan and denoted as MIROC5^41,42^.

### Statistical distribution of heatwaves

According to many studies^4,6,14,43^ a heatwave is defined as at least three consecutive days with daily temperature above the local 90th percentile threshold. Since heatwaves are extreme events, on average, they are not expected to occur every year. Supplementary Fig. 10 shows the number of years that do not show any heatwave in a decade of the present climate. We model the statistical distribution of heatwaves by applying a block maxima approach (see Coles 2002, Wehner 2018, Sterl 2008) with a block of 10-years. At each grid point, our set of data is composed by the maximum heatwave magnitude in 10-years. By fitting the maximum HWMId values in 10-year block with L-moments based estimators, we show that the decadal maxima of both HWMId and TX5x follows a Generalized Extreme Values distribution with skewness greater than or equal to zero^16^ (Freschet or Gumbel distribution, respectively. See Supplementary Fig. 11e–h). By using an Anderson Darling statistical test, suitable for extreme events because more weight is put on the tails (than in comparable tests such as Kolmogorov-Smirnov for instance), with the null hypothesis that decadal HWMId maxima are GEV, we demonstrate that the null hypothesis cannot be rejected in any location (*P*-value > 0.1 everywhere, see Supplementary Fig. 11a–d). This result is valid with all HAPPI models. By using the fitted GEV models we estimate 500 year heatwaves return levels (HW500Y) in the present climate and show the spatial distribution of the probability of occurrence of these values at 1.5 and 2 °C warming levels (Fig. 1). The same analysis is applied to the annual maximum of 5-day average of daily maximum temperature index (TX5x see Supplementary Fig. 12) for validation. The spatial distributions of HW500Y hazard calculated for the HWMId and the TX5x indices compare very well both in terms of pattern and probability values (Fig. 1 and Supplementary Fig. 12, respectively). Uncertainties associated to the occurrence of HW500Y are calculated as the 95^*th*^ confidence level of the GEV model fitted to the data (see Fig. 1a, Supplementary Figs. 13 and 14).

### Population density

To consider the impact of changes in heatwaves in populated regions of the world, we use a set of global, spatially explicit population projections that are consistent with the new Shared Socioeconomic Pathways (SSPs)^19^. The spatial population projections cover the period 2010–2100 in ten-year time steps. We have used population datasets at two different periods (2015 and 2075, decade 2010–2019 and 2070–2079, respectively) and under two different SSPs (SSP1 and SSP4). All population projections are remapped onto a regular grid of each HAPPI model by using a second order conservative remapping approach. Supplementary Fig. 15a shows population density in persons per km^2^ and normalized values (see section on normalization below), remapped on the MIROC5 model for all time periods and SSP pathways. All other models show the same maps.

### Human development index

As a proxy for vulnerability as a component of an illutstrative risk index (IRI) we use the Human Development Index (HDI), a composite indicator introduced by the UNDP in 1990 to assess the development of countries inspired by the concept of capabilities development by Amartya Sen^44^. HDI is based on the geometric average of three dimensions, all within suitable bounds: health (life expectancy at birth); education (expected and mean years of schooling), and standard of living (mean gross national income per capita, expressed in Purchasing Power Parity). In this study we define vulnerability as 1-HDI so that countries with the lowest HDI levels are associated with the highest vulnerability and vice-versa. As was done for the population data, we remap the most recent Human Development Index data (for the year 2015, see HDR2016) and projected HDI values under SSP1 and SSP4 pathways^26^ on the grid of each of the four HAPPI simulations used here (see Supplementary Fig. 15b for MIROC5 model). Because HDI data and projections are for country averages only, the approach taken here abstracts from the substantial heterogeneity in income, education, and health within countries, but captures the heterogeneity in HDI across countries.

### HDI versus other vulnerability indices

To evaluate the robustness of the HDI in accounting for vulnerability to climate we have estimated its correlation with the Notre Dame-Global Adaptation Initiative Country Index^25^ (ND-GAIN), a national index constructed from 45 indicators of vulnerability and readiness to respond to climate change in six sectors: food, water, health, ecosystem, services, human habitat, and infrastructure. In the present period, the HDI is significantly correlated with the ND-GAIN (Pearson correlation equal to 0.95 with a *p*-value < 0.001, see Supplementary Fig. 16) and this alternative index would thus produce a similar ranking of Countries if looking at their Economic Vulnerability to climate in the ND-GAIN. As ND-GAIN data projections are not available, we rely on HDI.

### Normalized population and human development index

For deriving the normalized version of IRI, and thus to illustrate composite spatial patterns of hazard, exposure, and vulnerability at the global scale, rather than definitive or quantitative risk estimates, the distribution of population density and HDI values are normalized by means of Johnson’s transformation^27^ (see Supplementary Fig. 17).  Normalization is needed to guarantee the homogeneity of the variances^45^ of the variables aggregated into the IRI. This illustrative approach implicitly assumes equal relative weights of exposure and vulnerability of the respective normalized distributions. Our normalization method consists of:

First: removing ties from population and (1-HDI) values for the present period (2015). Ties are removed only for statistical purposes in order to find the best statistical distribution fitting our data; they are not removed from risk maps. In fact, two locations with the same population density (or 1-HDI values) will have the same normalized score;

Second: fitting the present population density and (1-HDI) values with the Johnson Family curves^27^

Third: using the Cumulative Density distribution function fitted to present data to transform projected population and (1-HDI) values into a uniform probability interval [0, 1] (see Supplementary Fig. 17). Note that, since present HDI spatial data follow a bounded distribution, future HDI values that are out of the range of the present HDI values would not have a corresponding normalized value. In order to avoid this the Johnson fit is done by imposing the maximum HDI range that by definition is equal to (0, 1)^20^. The same limitation does not apply to population density data, since it follows a Log-Normal distribution with a domain in [0, + ∞].

The lowest entry in the population or 1-HDI data (population or HDI equal to zero) takes a normalized value equal to zero. In contrast, the highest entry (maximum population or 1-HDI values) takes a value equal to one or very close to one. Maps of population and HDI values are reported in Supplementary Fig. 15. The goodness of fit of the Johnson Family curves fitted to population and (1-HDI) data have been tested by means of a Kolmogorov-Smirnov test of hypothesis. In both cases we cannot reject the null hypothesis that population density and (1-HDI) datasets follow a Log-Normal and a Bounded distribution, respectively.

### Illustrative risk index at the global scale

At each location normalized IRI (expressed in %) is calculated as the product of the probability of occurrence of HW500Y multiplied by normalized population density and 1-HDI values:

1 $${\mathrm{IRI}} = ({\mathrm{HW}}_{{\mathrm{hazard}}} \times {\mathrm{Population}}_{{\mathrm{exposure}}} \times (1 - {\mathrm{HDI}})_{{\mathrm{vulnerability}}})\times100$$

with all components of the product above normalized in [0, 1].

### Code availability

Codes and additional information can be provided by directly contacting the authors.

## Supplementary Information

Supplementary Information

## Data Availability

All data used in analysis available in public repositories or upon request. Daily maximum temperature data are available at the following repository: http://portal.nersc.gov/c20c/data/. Population density data are available at: http://sedac.ciesin.columbia.edu/data/set/popdynamics-pop-projection-ssp-2010-2100. HDI data are reported in Supplementary Table 1 and are available upon request or from the repository reported in Crespo and Lutz^26^. The script used for calculating the HWMId is publicly available as a function of an R package extRemes (see: https://www.rdocumentation.org/packages/extRemes/versions/2.0-8/topics/hwmid).

## References

[1] Wehner, M. et al. Changes in extremely hot days under stabilized 1.5 °C and 2.0 °C global warming scenarios as simulated by the HAPPI multi-model ensemble. *Earth Syst. Dynam.* **9**, 299–311 (2018).

[2] Russo S, Sillmann J, Sterl A (2017). Humid heat waves at different warming levels. Sci. Rep..

[3] Lehner F (2017). Projected drought risk in 1.5 degrees C and 2 degrees C warmer climates. Geophys. Res. Lett..

[4] Sillmann J, Kharin VV, Zhang X, Zwiers FW, Bronaugh D (2013). Climate extremes indices in the CMIP5 multimodel ensemble: Part 2. Future climate projections. J. Geophys. Res..

[5] Fischer EM, Schär C (2010). Consistent geographical patterns of changes in high-impact European heatwaves. Nat. Geosci..

[6] Alexander, L. V. et al. Global observed changes in daily climate extremes of temperature and precipitation. *J. Geophys. Res*. **111**, D05109 (2006).

[7] Harrington LJ, Frame D, King AD, Otto FE (2018). How uneven are changes to impact‐relevant climate hazards in a 1.5 °C world and beyond?. Geophys. Res. Lett..

[8] King, A. D. & Harrington, L. J. The inequality of climate change from 1.5 °C to 2 °C of global warming. *Geophys. Res. Lett*. **45**, 4529-5228 (2018).

[9] IPCC. in *Climate Change 2014: Impacts, Adaptation, and Vulnerability. Part A: Global and Sectoral Aspects. Contribution of Working Group II to the Fifth Assessment Report of the Intergovernmental Panel on Climate Change* (eds Field, C. B. et al.) 1–32 (Cambridge University Press, Cambridge and New York, USA, 2014).

[10] Cardona, O. D. et al. in *Managing the Risks of Extreme Events and Disasters to Advance Climate Change Adaptation. A Special Report of Working Groups I and II of the Intergovernmental Panel on Climate Change (IPCC)* (eds Field, C. B. et al.) 65–108 (Cambridge University Press, Cambridge, and New York, USA, 2012).

[11] SREX, I. (2012). Special Report of Working Groups I and II of the Intergovernmental Panel on Climate Change (IPCC).

[12] Sillmann, J., Russo, S., Sippel, S. & Alnes, K. From hazard to risk. *Bull. Amer. Meteor. Soc.* **99**, 1689–1693 (2018).

[13] O’Neill BC (2014). A new scenario framework for climate change research: the concept of shared socioeconomic pathways. Clim. Change.

[14] Russo, S., Sillmann, J. & Fischer, E. M. Top ten European heat-waves since 1950 and their occurrence in the coming decades. *Environ. Res. Lett*. **10**, 124003 (2015)

[15] Sterl A (2008). When can we expect extremely high surface temperatures?. Geophys. Res. Lett..

[16] Coles, S. *An Introduction to Statistical Modeling of Extreme Values* (Springer, Berlin, 2001).

[17] Jones B, Tebaldi C, OŃeill BC, Keith Oleson k, Gao J (2018). Avoiding population exposure to heat-related extremes: demographic change vs climate change. Clim. Chang..

[18] Byers EA (2018). Global exposure and vulnerability to multi-sector development and climate change hotspots. Environ. Res. Lett..

[19] Jones B, O’Neill BC (2016). Spatially explicit global population scenarios consistent with the shared socioeconomic pathways. Environ. Res Lett..

[20] UNDP (United Nations Development Programme) (2016). Human Development Report 2016: Human Development for Everyone.

[21] Arent, D. J. et al. in *Climate Change 2014 Impacts, Adaptation and Vulnerability: Part A: Global and Sectoral Aspects* 659-708 (Cambridge University Press, 2015).

[22] Nordhaus W (2014). Estimates of the social cost of carbon: concepts and results from the DICE-2013R model and alternative approaches. J. Assoc. Environ. Resour. Econ..

[23] Tol RS (2009). The economic effects of climate change. J. Econ. Perspect..

[24] Füssel, H.-M. *Review and Quantitative Analysis of Indices of Climate Change Exposure, Adaptive Capacity, Sensitivity, and Impacts *(World Bank, Washington, 2009).

[25] Chen, C. et al. *University of Notre Dame Global Adaptation Index.**Country Index Technical Report* (University of Notre Dame, 2015).

[26] Crespo Cuaresma J, Lutz W (2016). The demography of human development and climate change vulnerability: a projection exercise. Vienna Yearbook of Population Research.

[27] Giovanardi F, Finoia MG, Russo S, Amori M, Di Lorenzo B (2006). Coastal waters monitoring data: frequency distributions of the principal water quality variables. J. Limnol..

[28] Harrington LJ (2016). Poorest countries experience earlier anthropogenic emergence of daily temperature extremes. Environ. Res. Lett..

[29] Fawcett AA (2015). Can Paris pledges avert severe climate change?. Science.

[30] Gasparrini A (2015). Mortality risk attributable to high and low ambient temperature: a multicountry observational study. Lancet.

[31] Gilleland E, Katz RW (2016). extremes 2.0: an extreme value analysis package in r. J. Stat. Softw..

[32] Sanderson BM (2017). Community climate simulations to assess avoided impacts in 1.5 °C and 2 °C futures. Geosci. Model Dev..

[33] Mitchell D (2017). Half a degree additional warming, projections, prognosis and impacts (happi): Background and experimental design. Geosci. Model Dev..

[34] Fischer EM, Beyerle U, Schleussner CF, King AD, Knutti R (2018). Biased Estimates of Changes in Climate Extremes From Prescribed SST Simulations. Geophys. Res. Lett..

[35] Barriopedro D (2011). The hot summer of 2010: Redrawing the temperature record map of Europe. Science.

[36] Barcikowska, M. J. et al. Euro-Atlantic winter storminess and precipitation extremes under 1.5 °C versus 2.0 °C warming scenarios. *Earth Syst. Dynam.* **9**, 679–699 (2017).

[37] von Salzen K (2013). The Canadian Fourth Generation Atmospheric Global Climate Model (CanAM4). Part I: Representation of Physical Processes. Atmosphere-Ocean.

[38] Neale, R. B. et al. *Description of the NCAR Community!Atmosphere Model (CAM4)*. NCAR Tech. Note NCAR/TN-485+STR (National Center for Atmospheric Research, Boulder, CO, 2011).

[39] Oleson, K. W. et al. *Technical Description of version 4.0 of the Community Land Model (CLM)*. NCAR Tech. Note NCAR/TN-478+STR (National Center for Atmospheric Research, Boulder, CO, 2010).

[40] Stevens B (2013). Atmospheric component of the mpi-m earth system model:echam6. Model. Earth Sys..

[41] Shiogama H (2013). An event attribution of the 2010 drought in the South Amazon region using the MIROC5 model. Atmos. Sci. Lett..

[42] Shiogama H (2014). Attribution of the june-july 2013 heat wave in the southwestern united states. SOLA.

[43] Frich P (2002). Global changes in climatic extremes during the 2nd half of the 20th century. Clim. Res..

[44] Sen A (1985). Well-being, agency and freedom: the Dewey lectures 1984. J. Philos..

[45] Pearson, K. *Mathematical Contributions to the Theory of Evolution. XIV. On the General Theory of Skew Correlation and Non-Linear Regression.**Drapers’Company Research Memoirs, Biometric Series II* (Cambridge University Press, 1905).

