# Supplementary material for: Half a degree and rapid socioeconomic development matter for heatwave risk
Source: Nat Commun. 2019 Jan 11;10:136. doi: 10.1038/s41467-018-08070-4 (PMC6329840; doi:10.1038/s41467-018-08070-4)
Supplement: Supplementary file 1 — Supplementary Information [file 41467_2018_8070_MOESM1_ESM.pdf]

Half a degree and rapid socioeconomic development matter for heatwave risk
Supplementary Information

Russo et. al.

Supplementary Figures

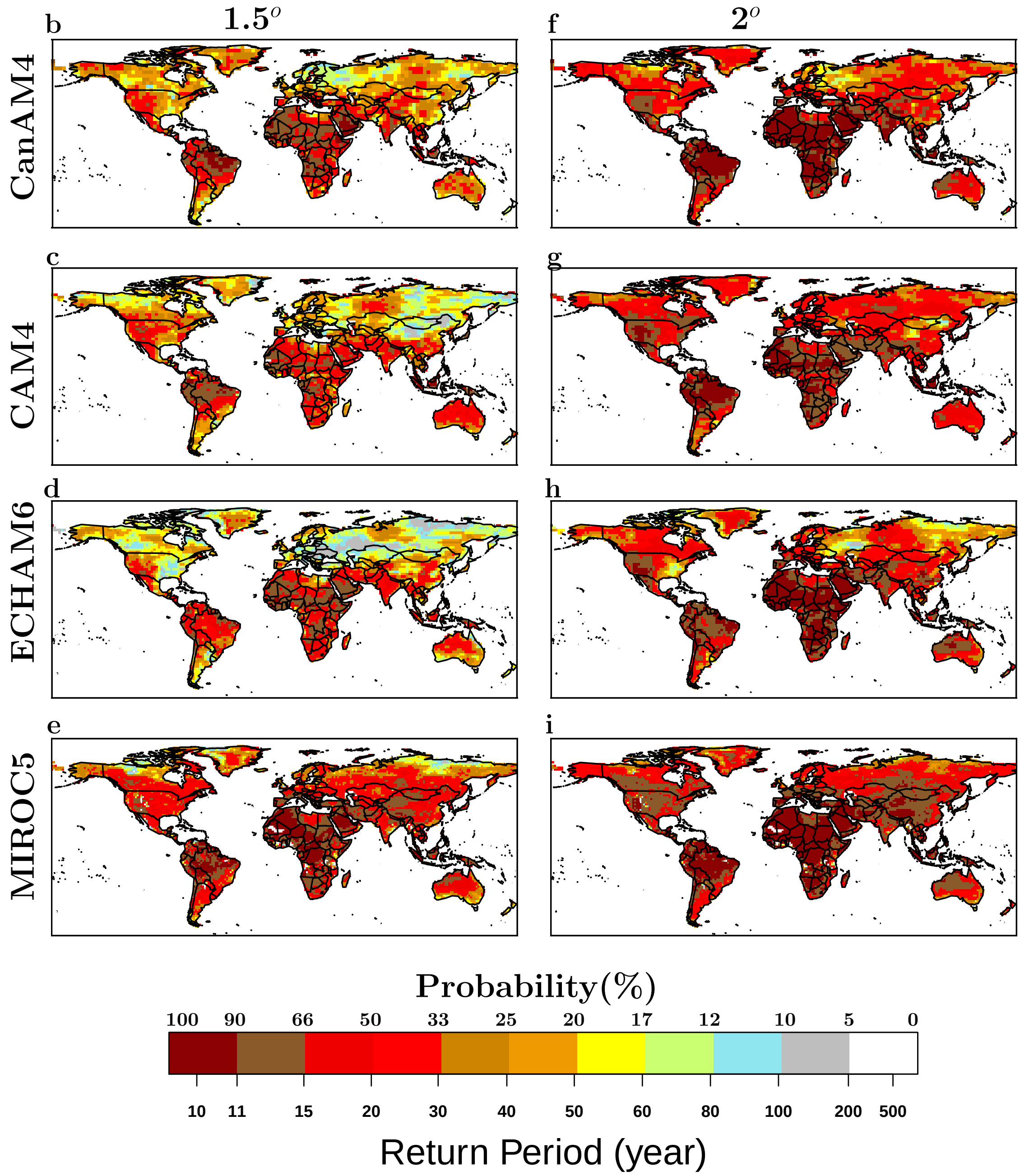

Supplementary Figure 1 — Probability of occurrence of extreme heatwave. Spatial distribution of the probability and return level of a heatwave with one-hundred years return period under 1.5^{o}C (b-e) and 2.0^{o}C (f-i) warming for multiple models.

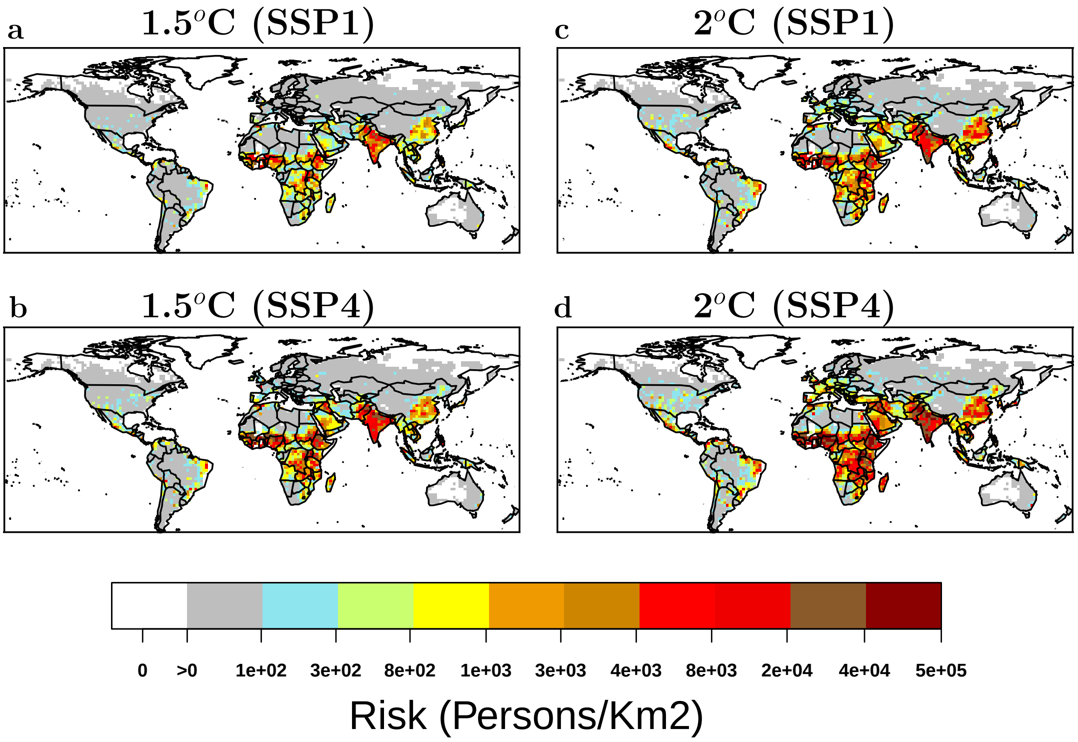

Supplementary Figure 2 — Risk map with non normalized population density. The risk at each grid point is calculated as the product of the probability of occurrence of HW500Y for the values of population density and for normalized value of Human Development Index for the ECHAM6 model. The dimension in colour scale represents the value of population density scaled by normalized measures of hazard and vulnerability.

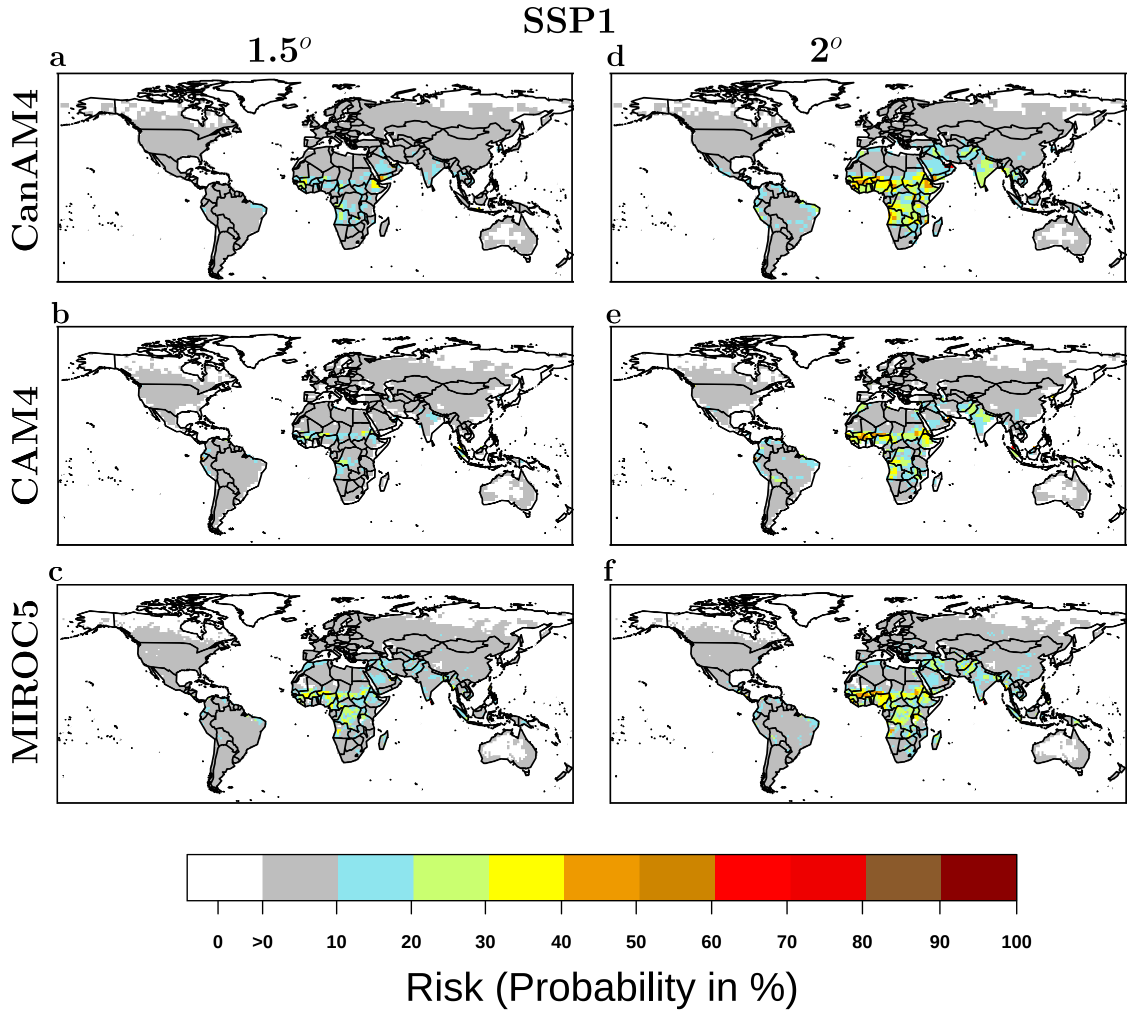

Supplementary Figure 3 — Heatwave Risk under the low vulnerability pathway. The risk at each grid point is calculated as the product of the probability of occurrence of HW500Y for the normalized values of population density and Human Developement Index.

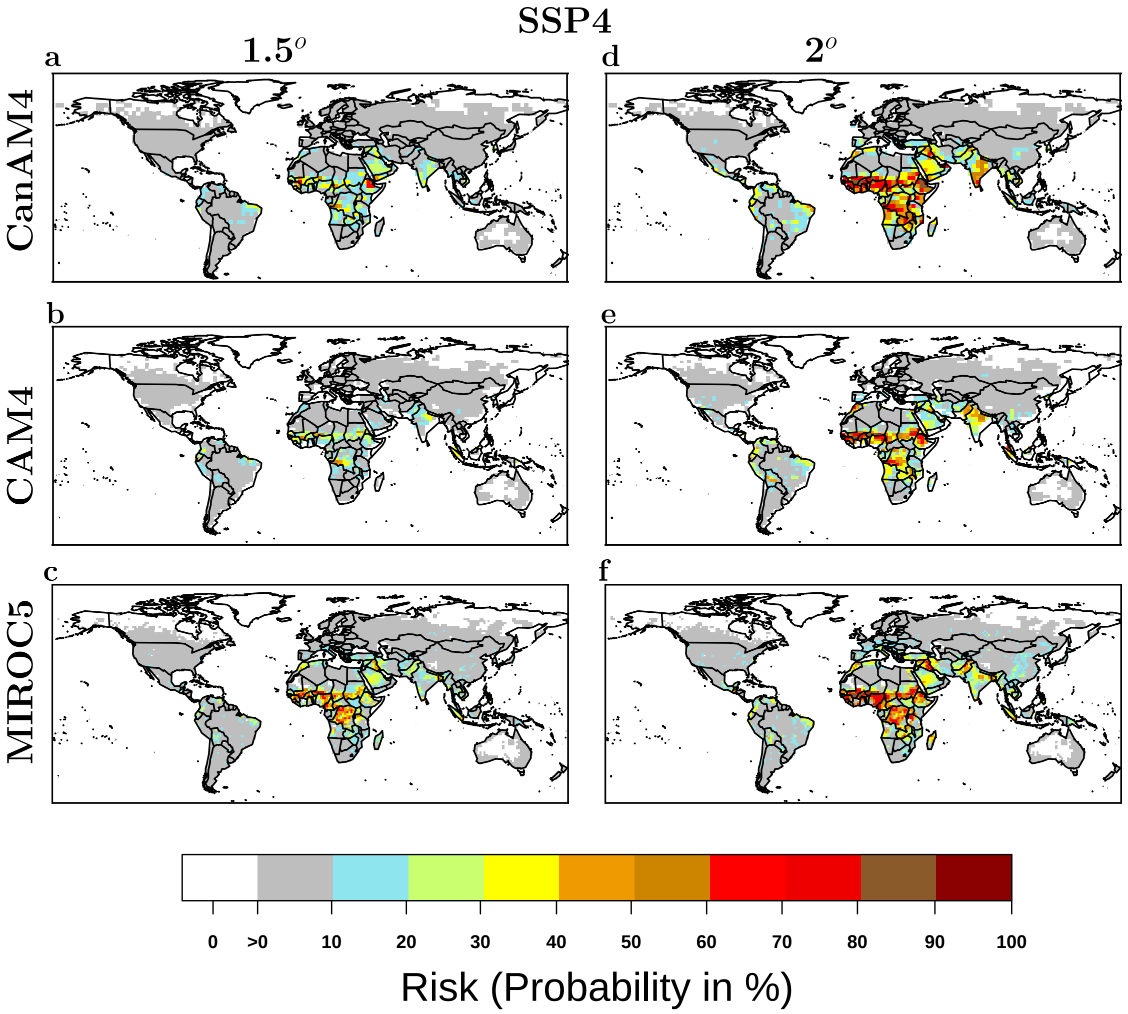

Supplementary Figure 4 — Heatwave Risk under the high vulnerability pathway. The risk at each grid point is calculated as the product of the probability of occurrence of HW500Y for the normalized values of population density and Human Developement Index.

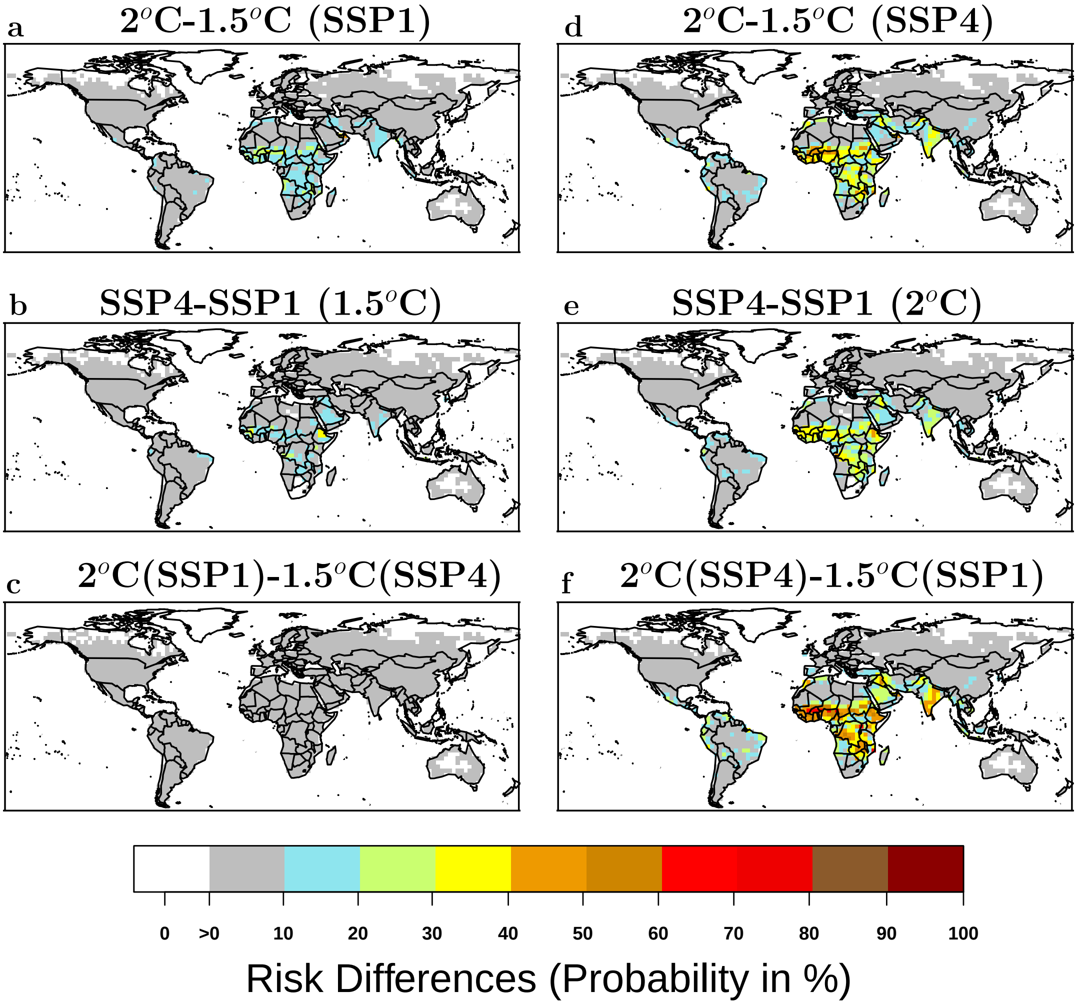

Supplementary Figure 5 — Differences in heatwave Risk for the CanAM4 model. The risk differences are calculated at each grid point by means of risk values. **a,d** Differences of heatwave risk between 2^oC and 1.5^{o}C under the low vulnerability (SSP1) and high vulnerability (SSP4) scenarios, respectively. **b,e** Differences of heatwave risk between SSP4 and SSP1 1.5^{o}C and 2^oC warming levels, respectively. **c,** Differences of heatwave risk between 2^oC under the SSP1 scenario and 1.5^{o}C under the SSP4 scenarios. **c,** Differences of heatwave risk between the most pessimistic (2^oC level of warming under the SSP4 scenario) and most optimistic (1.5^{o}C level of warming under the SSP1 scenario) expected scenarios.

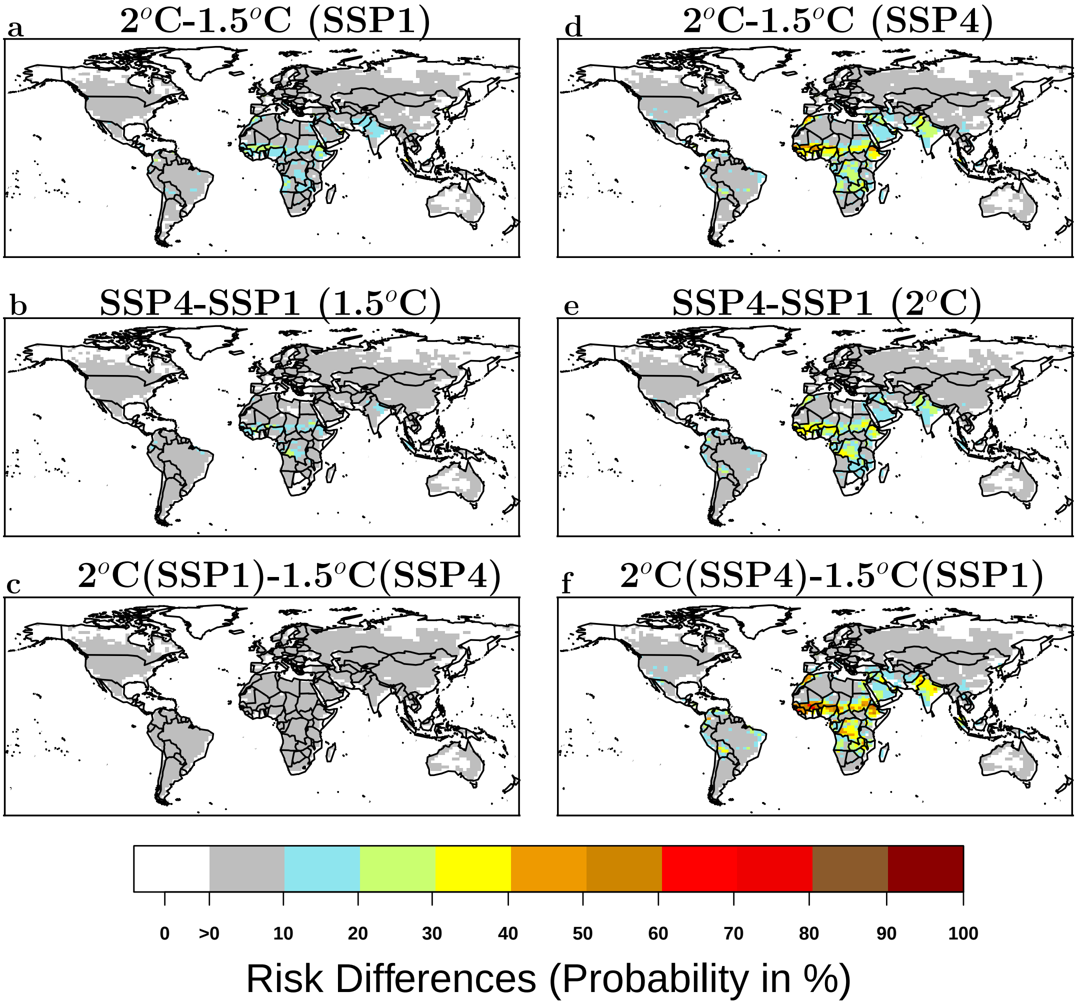

Supplementary Figure 6 — Differences in heatwave Risk for the CAM4 model. The risk differences are calculated at each grid point by means of risk values. **a,d** Differences of heatwave risk between 2°C and 1.5^{o}C under the low vulnerability (SSP1) and high vulnerability (SSP4) scenarios, respectively. **b,e** Differences of heatwave risk between SSP4 and SSP1 1.5^{o}C and 2°C warming levels, respectively. **c,** Differences of heatwave risk between 2°C under the SSP1 scenario and 1.5^{o}C under the SSP4 scenarios. **c,** Differences of heatwave risk between the most pessimistic (2°C level of warming under the SSP4 scenario) and most optimistic (1.5^{o}C level of warming under the SSP1 scenario) expected scenarios.

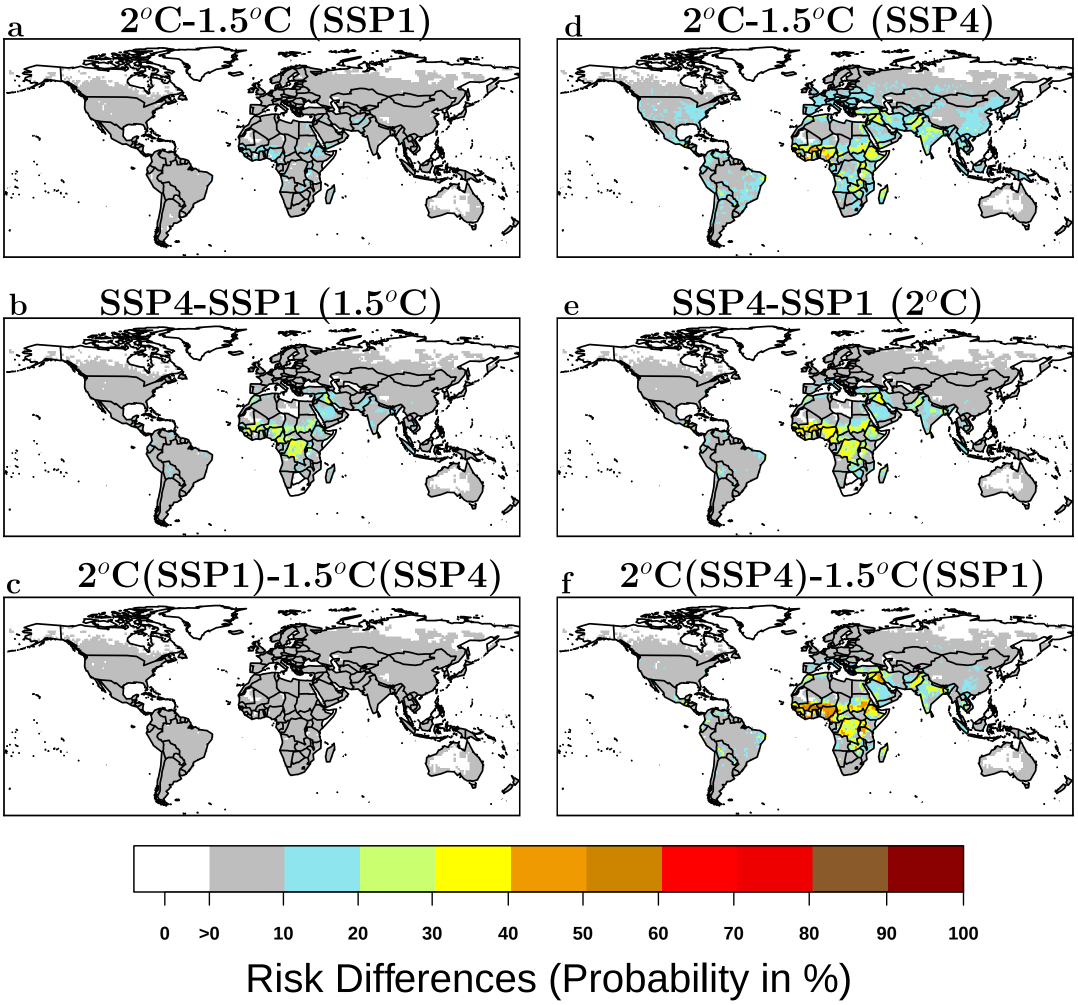

Supplementary Figure 7 — Differences in heatwave Risk for the MIROC5 model. The risk differences are calculated at each grid point by means of risk values. **a,d** Differences of heatwave risk between 2°C and 1.5^{o}C under the low vulnerability (SSP1) and high vulnerability (SSP4) scenarios, respectively. **b,e** Differences of heatwave risk between SSP4 and SSP1 1.5^{o}C and 2°C warming levels, respectively. **c,** Differences of heatwave risk between 2°C under the SSP1 scenario and 1.5^{o}C under the SSP4 scenarios. **c,** Differences of heatwave risk between the most pessimistic (2°C level of warming under the SSP4 scenario) and most optimistic (1.5^{o}C level of warming under the SSP1 scenario) expected scenarios.

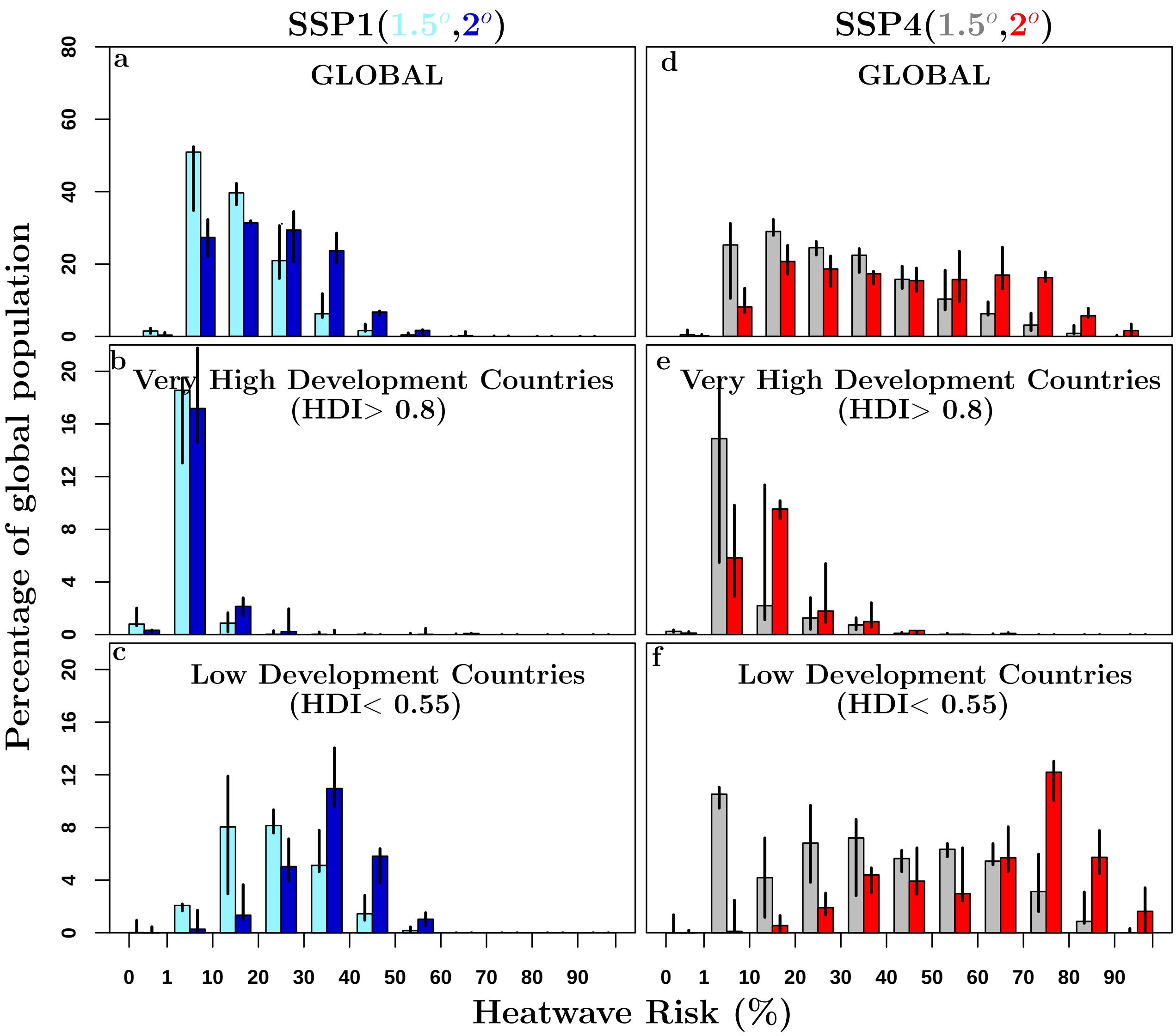

Supplementary Figure 8 — Population as function of IRI with HW100Y hazard. a, Bar plots show the ensemble model median of the fraction of global population at different heatwave hazards at 1.5^o(gray bars) and 2^o(red bars) warming. The bar plot are calculated for all the grid points of the global domain with population density greater than 0. **b,c** as a, but for very high and low human development countries with HDI>0.8 and HDI<0.55, respetively.

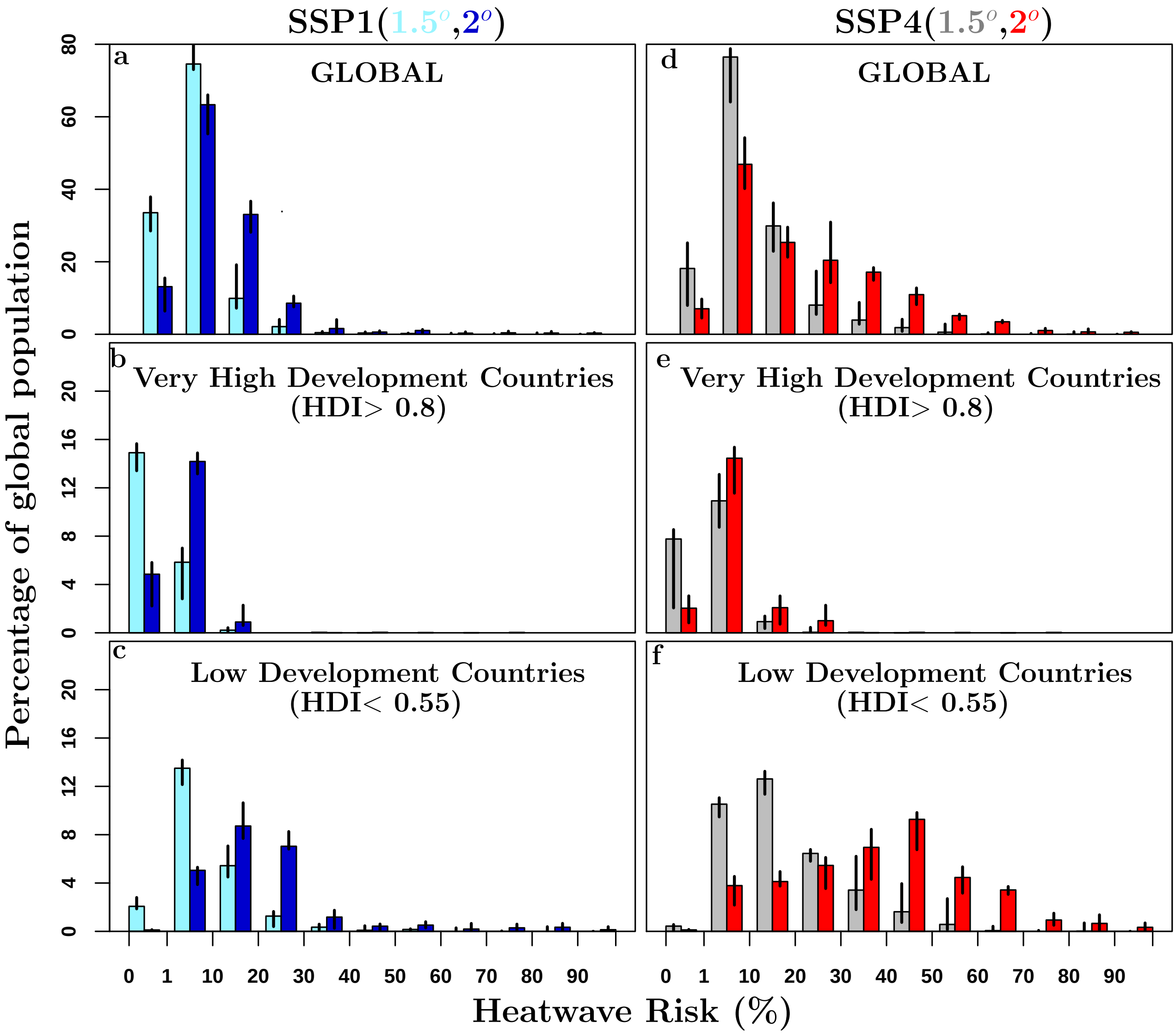

Supplementary Figure 9 — Population as function of IRI with no-normalized HDI values. a, Bar plots show the ensemble model median, with associated range represented by black lines, of the fraction of global population at different heatwave risk levels at 1.5^oC(gray bars) and 2^oC(red bars) warming, and under the SSP1 scenario. The bar plots are calculated for all the grid points of the global domain with population density greater than 0. **b,c** as a, but for countries with HDI>0.8 and HDI<0.55, corresponding to very high and low human development countries, respectively (see Fig. S4). **d,e,f** as a,b, and c, respectively, but for the SPP4 pathway.

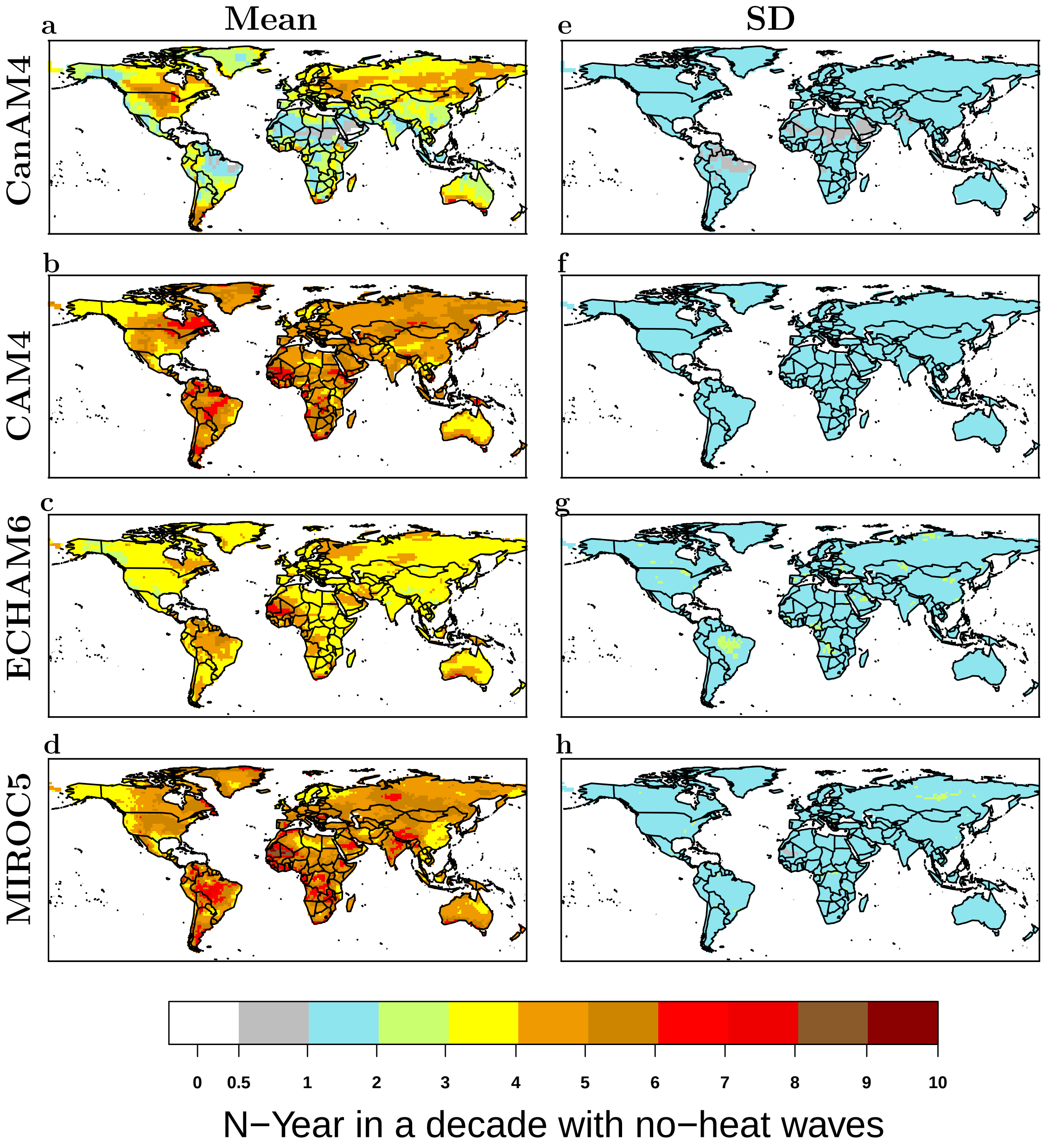

Supplementary Figure 10 — Spatial Number of years in a decade block with no-heat waves a, Mean of the number of years that in a decade have zero heat waves. **b,** As a, but for Standard Deviation.

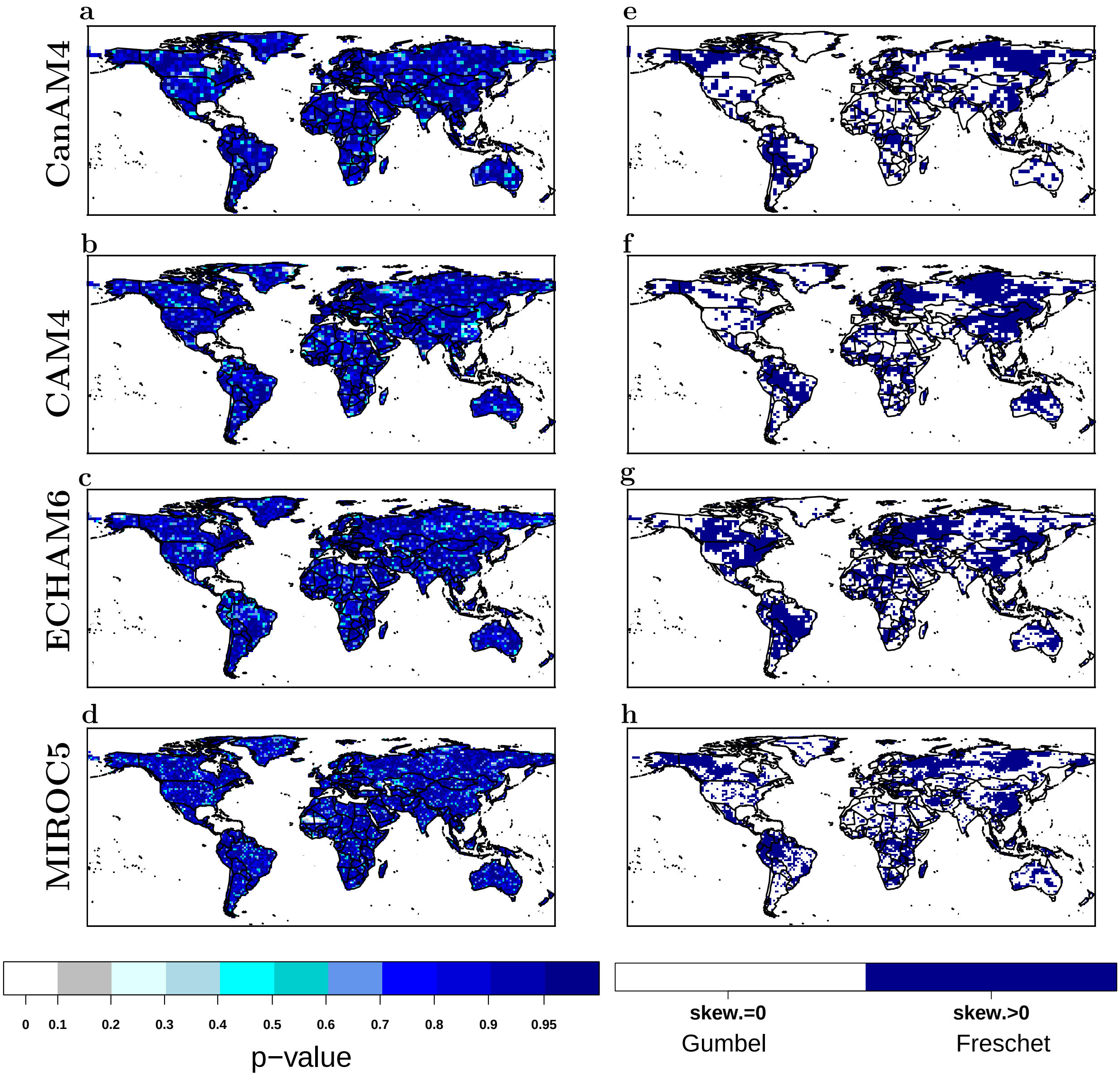

Supplementary Figure 11 — The statistical distribution of heatwaves. p-value and Generalized Extreme Value distribution type of the GEV model fitted to HWMId HAPPI data for the historical period.

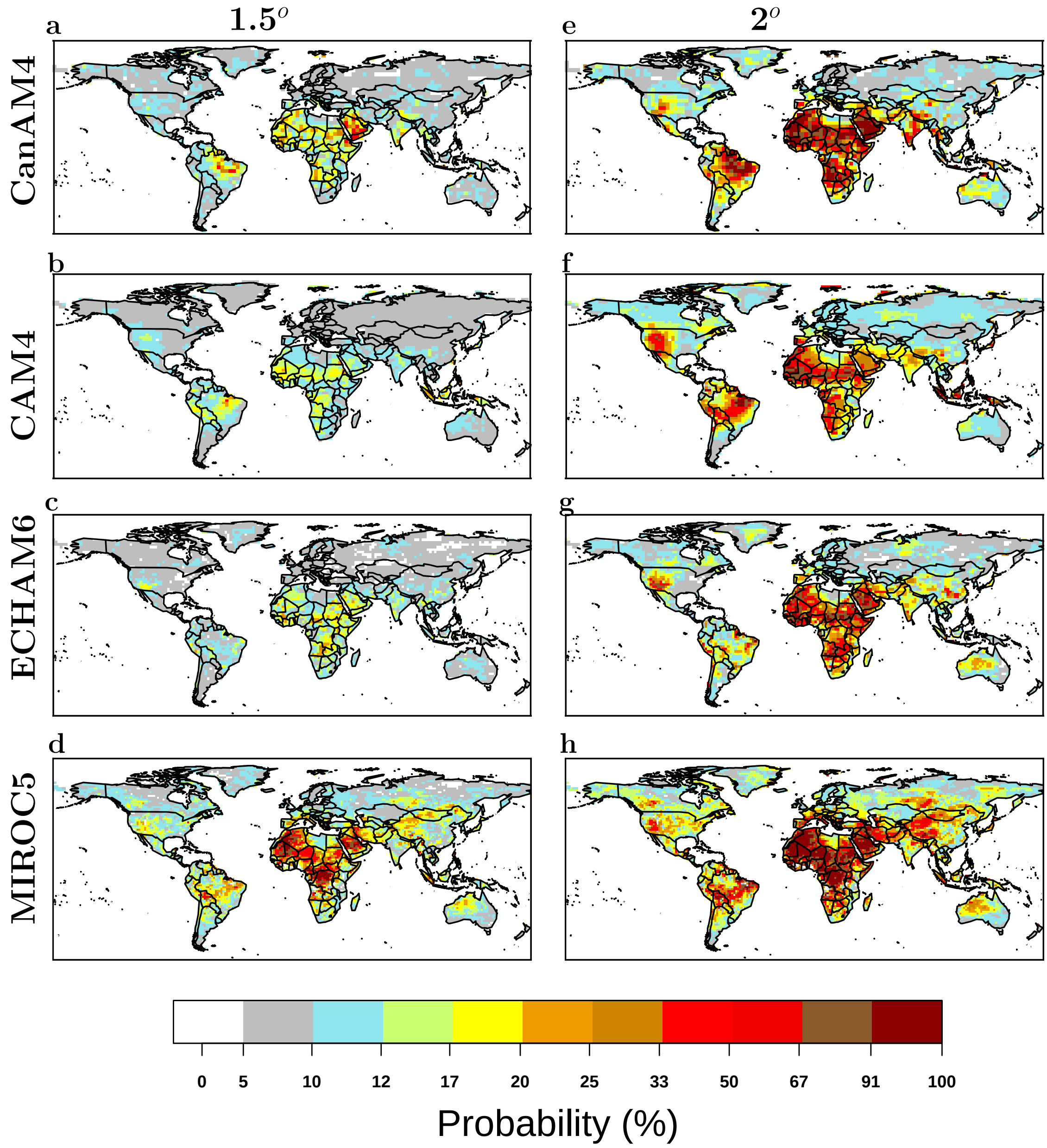

Supplementary Figure 12 — Extreme heatwaves with TX5x index. Probability of occurrence and associated return levels of extreme heatwave (HW500Y) at different warming levels with the TX5x decadal maxima

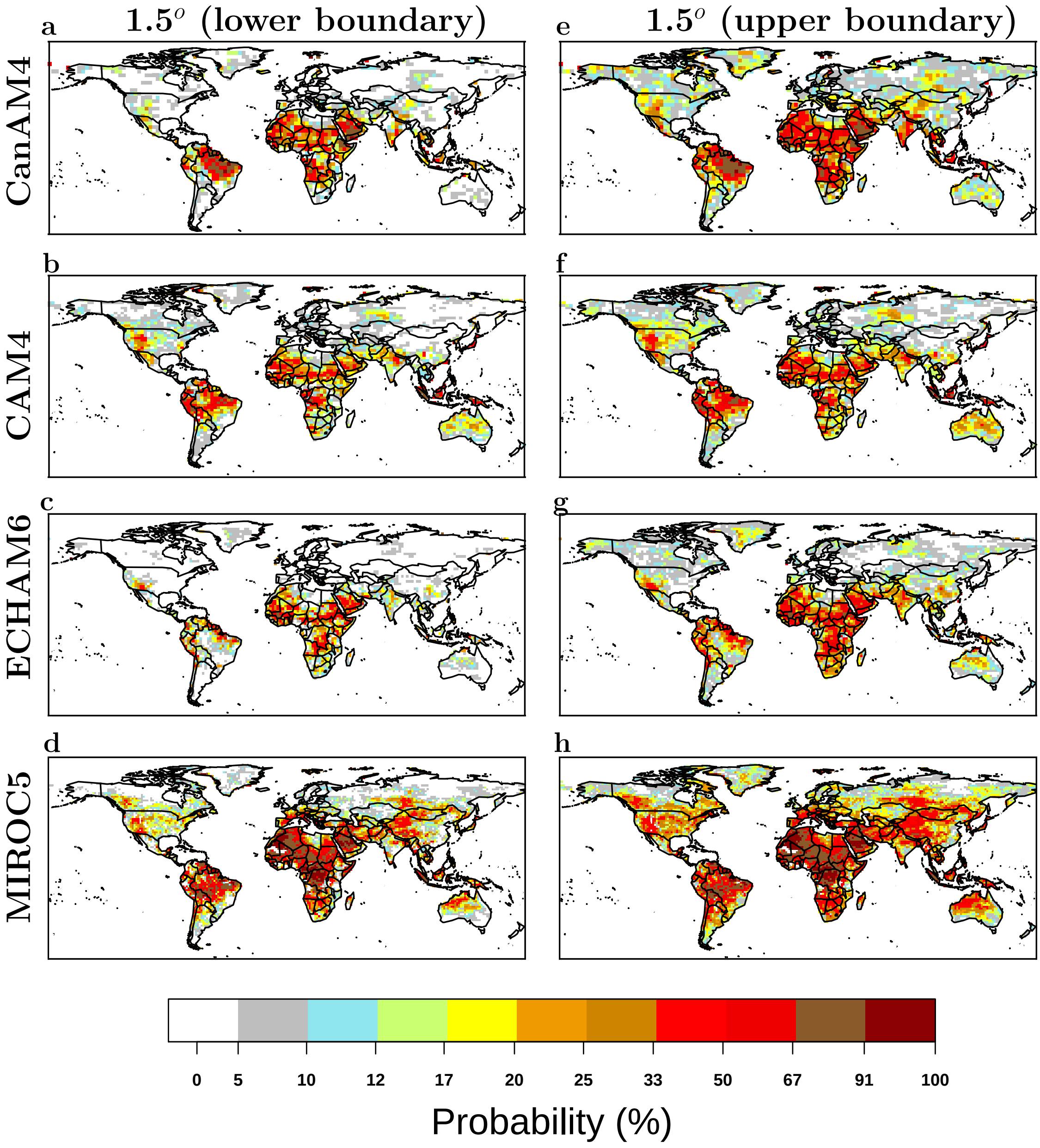

Supplementary Figure 13 — Confidence levels of heatwave risk estimation. Spatial distribution of upper and lower 95th confidence level of the GEV fits to the decadal HWMId maxima at a warming level of 1.5^oC

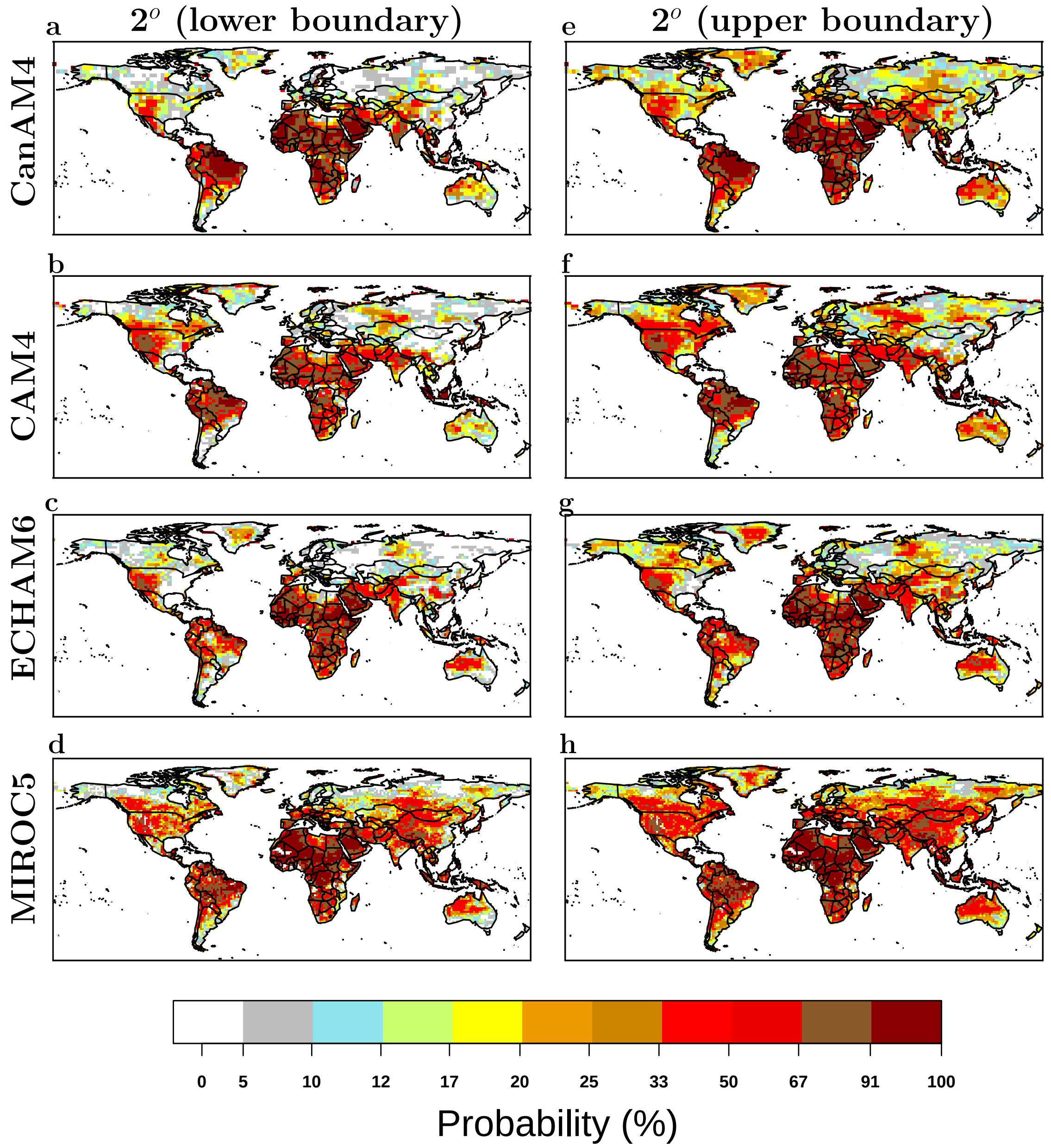

Supplementary Figure 14 — Confidence levels of heatwave risk estimation. Spatial distribution of upper and lower 95th confidence level of the GEV fits to the decadal HWMId maxima at a warming level of 2^oC.

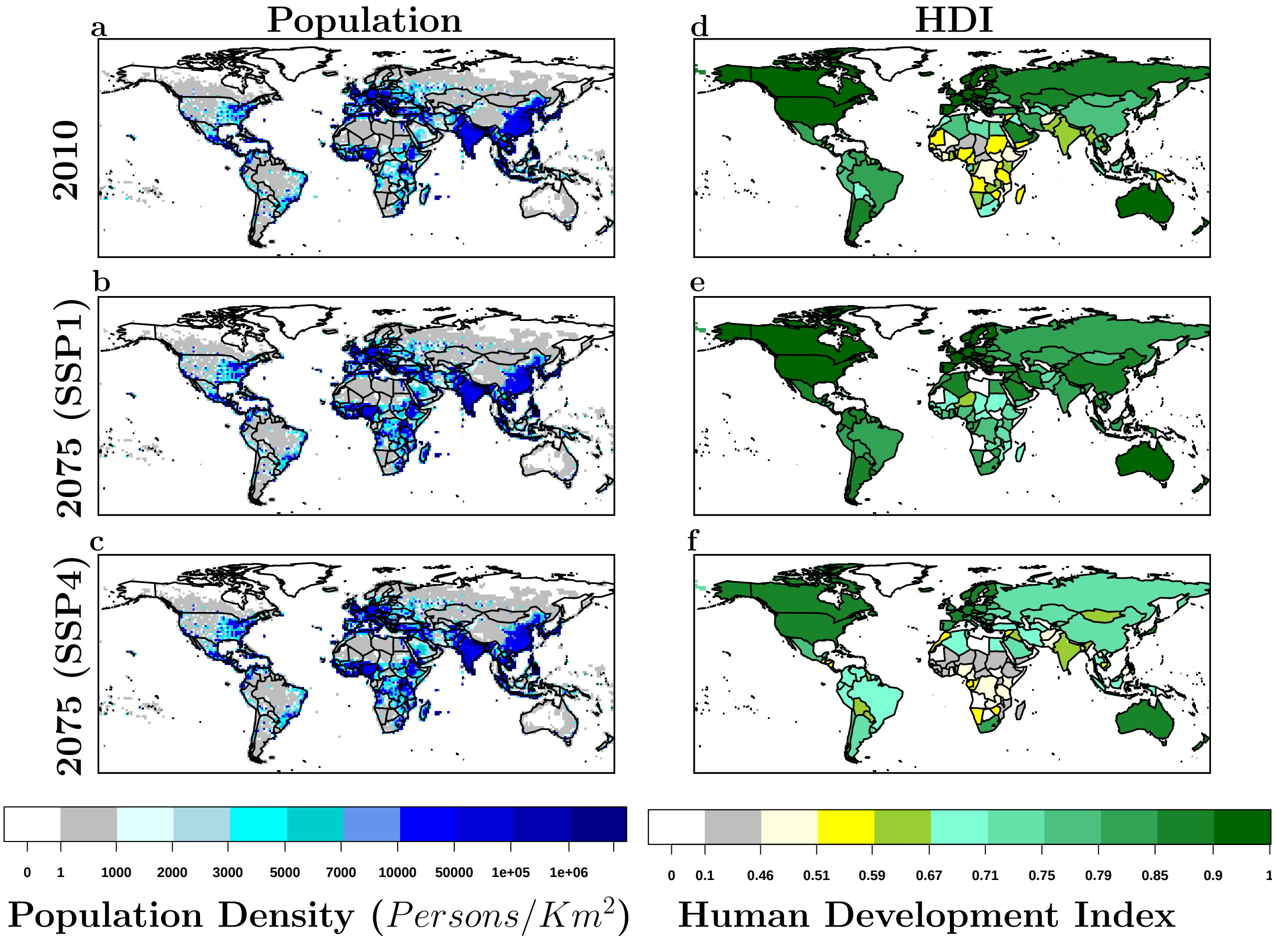

Supplementary Figure 15 — Maps of Population density and Human Development Index a, Population Density in persons/Km^2 interpolated on the MIROC5 model grid. **b,c** As a, but for Projected population in 2075 (decade 2070-2079) under the SSP1 and SSP4 socio economic pathways, respectively. **d,e,f** As a,b,c but for the Human Development Index in 2075.

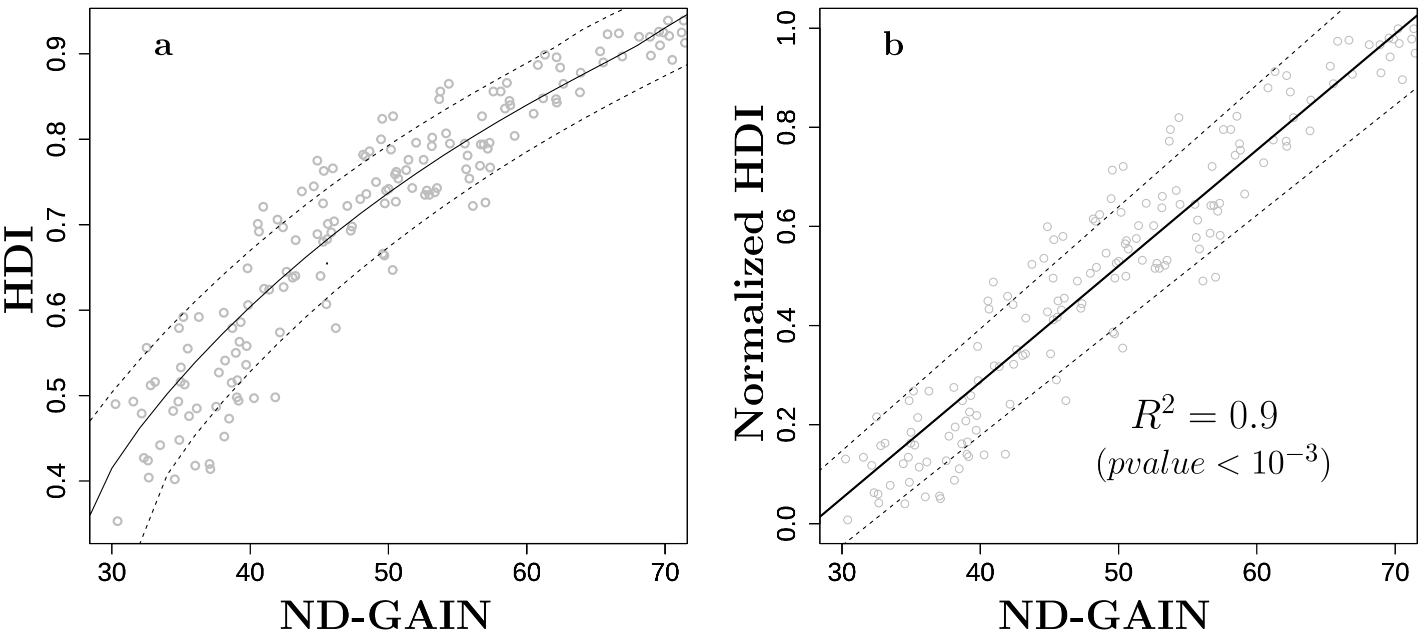

Supplementary Figure 16 — Scatterplot and relationship between HDI and ND-GAIN. a, HDI (y-axis) versus ND-GAIN (x-axis). **b,** as a, but for normalized HDI values. Black continuous and dashed lines represent the linear relationship and the corresponding level of confidence at 95% obtained by means of a linear regression between the Normalized HDI values and ND-GAIN values. The black contineous and dashed lines in a are obtained by the linear lines in b applied to no normalized HDI values.

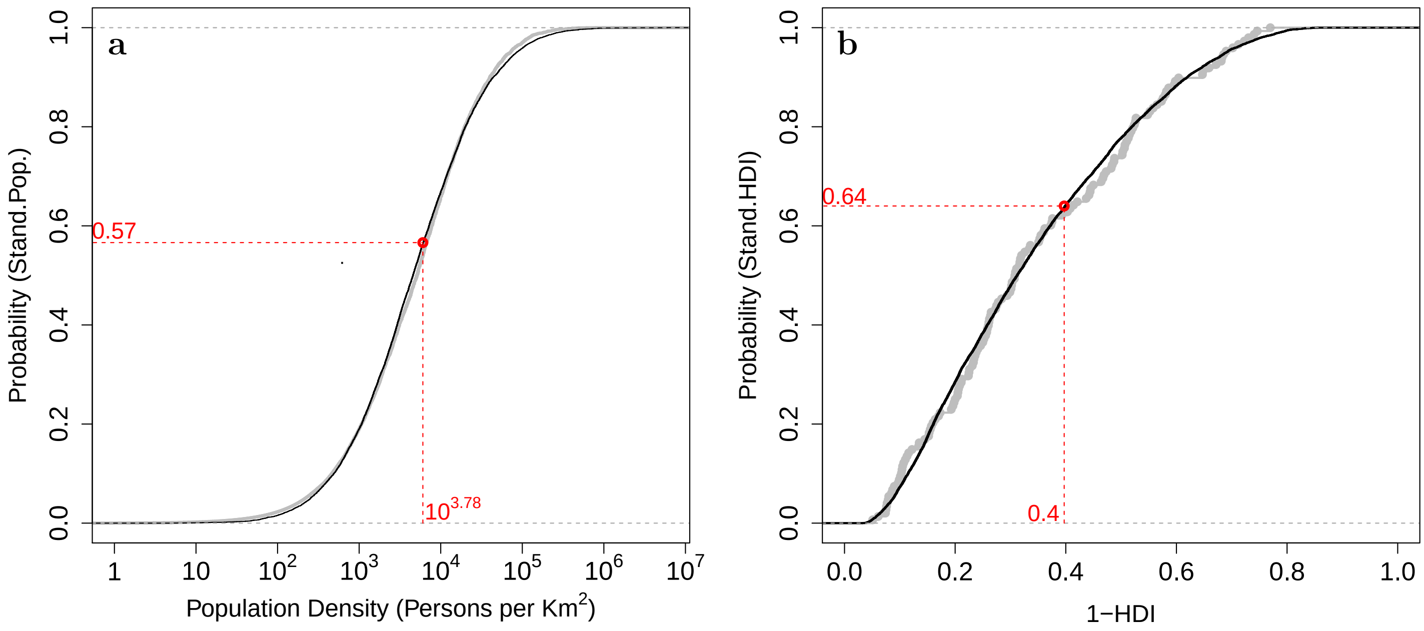

Supplementary Figure 17 — Normalization of population density and Human Development Index. a, normalization of population density (x-axis) in standard uniform units (y-axis). The black curves represents the Johnson's Cumulative Density Function (see methods) fitted to the global density population values (gray filled circles) over the present period (2010). The red open circle represents a value of future population ($10^{3.78}$ in x-axis) and the corresponding normalized value (0.57 in y-axis). **b,** as a, but for (1-HDI) values.

Supplementary Tables

**VERY HIGH Hum. Dev.**	2015	2075 (SSP1)	2075 (SSP4)	**LOW Hum. Dev.**	2015	2075 (SSP1)	2075 (SSP4)
Norway	0.949	0.908	0.835	Swaziland	0.541	0.739	0.478
Australia	0.939	0.902	0.831	Syrian Arab Republic	0.536	0.770	0.515
Switzerland	0.939	0.891	0.808	Angola	0.533	0.738	0.477
Germany	0.926	0.882	0.806	United Republic of Tanzania	0.531	0.699	0.403
Denmark	0.925	0.8464	0.762	Nigeria	0.527	0.733	0.466
Singapore	0.925	0.848	0.782	Cameroon	0.518	0.729	0.411
Netherlands	0.924	0.880	0.784	Papua New Guinea	0.516	0.692	0.269
Ireland	0.923	0.894	0.821	Zimbabwe	0.516	0.765	0.501
Iceland	0.921	0.894	0.821	Solomon Islands	0.515	0.764	0.501
Canada	0.920	0.886	0.812	Mauritania	0.513	0.763	0.500
United States	0.920	0.866	0.790	Madagascar	0.512	0.656	0.386
Hong Kong, China (SAR)	0.917	0.913	0.837	Rwanda	0.498	0.667	0.328
New Zealand	0.915	0.879	0.796	Comoros	0.497	0.684	0.362
Sweden	0.913	0.892	0.819	Lesotho	0.497	0.697	0.409
Liechtenstein	0.912	0.898	0.803	Senegal	0.494	0.695	0.341
United Kingdom	0.909	0.905	0.788	Haiti	0.493	0.739	0.450
Japan	0.903	0.882	0.815	Uganda	0.493	0.699	0.368
Republic of Korea	0.901	0.860	0.794	Sudan	0.490	0.673	0.370
Israel	0.899	0.826	0.729	Togo	0.487	0.685	0.375
Luxembourg	0.898	0.915	0.804	Benin	0.485	0.698	0.379
France	0.897	0.891	0.806	Yemen	0.482	0.697	0.390
Belgium	0.896	0.898	0.808	Afghanistan	0.479	0.696	0.400
Finland	0.895	0.866	0.797	Malawi	0.476	0.695	0.420
Austria	0.893	0.902	0.831	Côte dIvoire	0.474	0.716	0.365
Slovenia	0.890	0.845	0.762	Djibouti	0.473	0.714	0.488
Italy	0.887	0.886	0.782	Gambia	0.452	0.713	0.412
Spain	0.884	0.875	0.774	Ethiopia	0.448	0.695	0.368
Czech Republic	0.878	0.841	0.760	Mali	0.442	0.654	0.230
Greece	0.866	0.865	0.766	Liberia	0.427	0.680	0.277
Brunei Darussalam	0.865	0.846	0.764	Democratic Republic of the Congo	0.425	0.733	0.436
Estonia	0.865	0.821	0.743	Guinea-Bissau	0.424	0.660	0.319
Cyprus	0.856	0.881	0.803	Eritrea	0.420	0.660	0.315
Malta	0.856	0.886	0.815	Sierra Leone	0.420	0.661	0.310
Qatar	0.856	0.885	0.804	South Sudan	0.418	0.673	0.370
Poland	0.855	0.838	0.755	Guinea	0.414	0.692	0.269
Lithuania	0.848	0.826	0.751	Burundi	0.404	0.655	0.317
Chile	0.847	0.839	0.729	Burkina Faso	0.402	0.671	0.253
Saudi Arabia	0.847	0.814	0.695	Chad	0396	0.654	0.287
Slovakia	0.845	0.834	0.751	Niger	0.353	0.625	0.259
Portugal	0.843	0.863	0.735	Central African Republic	0.352	0.674	0.297
United Arab Emirates	0.840	0.839	0.727				
Hungary	0.836	0.815	0.719				
Latvia	0.830	0.822	0.745				
Argentina	0.827	0.825	0.683				
Croatia	0.827	0.820	0.738				
Bahrain	0.824	0.827	0.722				
Montenegro	0.807	0.815	0.750				
Russian Federation	0.804	0.778	0.698				
Romania	0.802	0.813	0.714				
Kuwait	0.800	0.824	0.728				

Supplementary Table 1 — List of very high and low human developed countries according to the 2015 HDI values and projected value for the year 2075 under SSP1 and SSP4 socio-economic pathways. (2015 data are taken from the Human Development Report 2016).
